# Minoxidil and nebivolol restore aortic elastic fiber homeostasis in diabetic mice via potassium channel activation

**DOI:** 10.3389/fphys.2025.1648727

**Published:** 2025-09-18

**Authors:** Auberi Henry, Laetitia Vanalderwiert, Floriane Oszust, Amandine Wahart, Daniel A. Carvajal Berrio, Eva M. Brauchle, Katja Schenke-Layland, Juergen Brinckmann, Heiko Steenbock, Laurent Debelle, Isabelle Six, Gilles Faury, Stéphane Jaisson, Philippe Gillery, Vincent Durlarch, Hervé Sartelet, Pascal Maurice, Amar Bennasroune, Laurent Martiny, Laurent Duca, Béatrice Romier, Sébastien Blaise

**Affiliations:** ^1^ UMR CNRS 7369 MEDyC, University of Reims Champagne-Ardenne, Reims, France; ^2^ Institute of Biomedical Engineering, Department for Medical Technologies and Regenerative Medicine, Eberhard Karls University Tübingen, Tübingen, Germany; ^3^ NMI Natural and Medical Sciences Institute, Reutlingen, Germany; ^4^ Department of Medicine/Cardiology, Cardiovascular Research Laboratories, David Geffen School of Medicine at UCLA, Los Angeles, CA, United States; ^5^ Department of Dermatology and Institute of Virology and Cell Biology, University of Lübeck, Lübeck, Germany; ^6^ Research Unit 7517, Pathophysiological Mechanisms and Consequences of Cardiovascular Calcifications (MP3CV), University of Picardie Jules Verne, Amiens, France; ^7^ University of Grenoble Alpes, INSERM, CHU Grenoble Alpes, Grenoble, France; ^8^ Reims Hospital, Biochemistry Department, Reims, France; ^9^ Cardiovascular and Thoracic Division, Reims Hospital, Reims, France

**Keywords:** elastogenesis, elastolysis, aging, diabetes, FOXO1, potassium channel

## Abstract

**Background:**

Diabetic patients experience a significant reduction in life expectancy, primarily due to early cardiovascular complications. A key feature is the premature degradation of elastic fibers (EFs), contributing to vascular stiffness.

**Objective:**

This study evaluates the capacity of two antihypertensive agents, minoxidil (a KATP channel opener) and nebivolol (a β-blocker with KATP activity), to restore EF homeostasis and arterial elasticity in diabetic mice.

**Methods:**

Mice are treated with two antihypertensive agents: minoxidil (an ATP-sensitive potassium (KATP) channel opener) or nebivolol (a β-blocker also active on KATP channels). The degree of wear and functionality of EF are assessed after these treatments. We complement this analysis by identifying molecular actors from smooth muscle cell cultures.

**Results:**

Our data show that by applying these antihypertensive agents in cultured vascular smooth muscle cells *in vitro* and in diabetic mice, we efficiently stimulate elastogenesis and inhibit elastolysis. Therefore, treatments restore functional EFs and limit their degradation. This brings blood pressure values of diseased mice close to normal ones (as in unaffected mice). Elastogenesis pathway stimulation and elastolysis inhibition are induced by the opening of sensitive KATP channels and the regulation of the forkhead box transcription factor (FOXO1).

**Conclusion:**

Minoxidil and nebivolol restore EF integrity and limit vascular aging in diabetic mice via K+ channel opening and FOXO1 repression. These findings highlight potassium channel–FOXO1 signaling as a therapeutic axis to counteract diabetic vascular complications.

## Introduction

According to the International Diabetes Federation and the World Health Organization, one in ten people worldwide has diabetes. Because prevalence has followed exponential evolution for several decades, estimations from these organizations suggest that 784 million people will be affected by diabetes in 2045 (Hu et al., 2015) The principal problem is that diabetes damage is usually irreversible; only prevention of its complications is currently possible.

Vascular diseases (hypertension, atherosclerosis, coronary heart disease, stroke, etc.) are the major complications of diabetes and are the leading cause of death for these patients. Conventionally, these vascular pathologies develop in aging people (around 70 years old), whereas, in patients with diabetes, they can be developed at an early age (around 55 years old) (Fox et al., 2004). Recent studies (Fontaine et al., 2003; Newman, 2015; Preston et al., 2018; Tam et al., 2020). Have shown that contracting diabetes is tantamount to premature age by approximately 15 years, thus explaining the increased risk of suffering from vascular disorders earlier in life. Several studies have highlighted that metabolic disorders such as diabetes and obesity not only reduce life expectancy but also mimic and accelerate biological aging processes (Nunan et al., 2022; Selle et al., 2023; Tam et al., 2020; Xu et al., 2021). This phenomenon, often referred to as “metabolic premature aging,” involves the disruption of key cellular mechanisms, including autophagy, increased apoptosis, telomere attrition, mitochondrial dysfunction, and cellular senescence. In parallel, extracellular matrix remodeling through excessive proteolysis further contributes to tissue degeneration and vascular dysfunction. These alterations resemble those observed during physiological aging and explain why diabetes is now considered a progeroid condition, particularly with respect to the vascular system. Our previous work (Vanalderwiert et al., 2024b) has demonstrated that db/db mice, beyond displaying hyperglycemia, develop aortic wall changes analogous to those found in aged non-diabetic animals, including elastic fiber fragmentation and stiffening, supporting their relevance as a model of accelerated vascular aging. The premature death observed during diabetes and/or obesity progressions, could be also associated, in part or in full, by the emergence of cellular processes characteristic of tissue aging, such as autophagy, apoptosis, telomere shortening, and senescence (Nunan et al., 2022; Selle et al., 2023; Tam et al., 2020; Xu et al., 2021), as well as extracellular processes including proteolysis (Burton and Faragher, 2018; Romier et al., 2018). Therefore, the development of pharmacotherapy strategies is needed to prevent diabetes-induced vascular complications. This statement is supported by lessons from the COVID-19 pandemic, which revealed that patients suffering from chronic pathologies, such as diabetes, are extremely vulnerable to the environment, including infectious stresses (Rico-Martin et al., 2021; Sanoudou et al., 2022).

Among possible preventive therapeutic strategies, the maintenance of a coherent and functional architecture of the vascular extracellular matrix (ECM) is of major importance (Baud et al., 2013). Indeed, we have recently demonstrated that the wall of the descending aorta in *db/db* mice exhibits cellular senescence coupled with premature aging of the ECM, similar to what is observed in aged mice (Vanalderwiert et al., 2024b). These anatomical alterations could explain the early onset of dysfunctions such as aortic stiffness and hypertension. Therefore, the mechanical properties of the ECM depend on its organization and composition. Indeed, a high proportion of collagen—responsible for strength and stiffness—and elastin—responsible for elasticity and resilience—allows the formation of a dense fibrillar network that is resistant to high extensions. Elastin is particularly present in tissues subjected to mechanical stress, such as arteries, skin, and lungs. Conversely, proteoglycans and glycoproteins form a looser network capable of withstanding compressive stress. At the vascular level, the high proportion of collagen and elastin molecules in the wall allows large arteries (i.e., elastic arteries) to resist to stretch caused by systolic pressure (Karamanos et al., 2021) and to smooth the discontinuous blood pressure and flow induced by the heart, namely, the Windkessel effect (Wagenseil and Mecham, 2009). Maintenance of arterial ECM ensures proper arterial function and is essential. It is mainly carried out by fibroblasts and/or smooth muscle cells (SMCs) that can both synthesize and remodel ECM.

**FIGURE 1:**
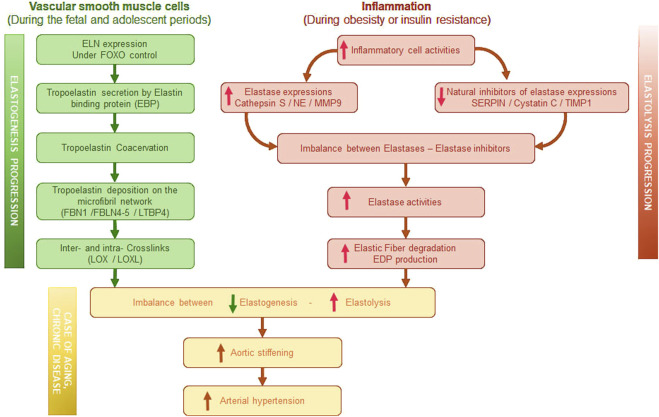
Graphical summary of elastogenesis progression induced in vascular smooth muscle cells and of elastolysis progression induced by inflammation. EBP, Elastin binding protein–FBN, Fibrilin–FBLN, Fibulin–LTBP, latent transforming growth factor beta binding protein–LOX, Lysyl-oxidase–LOXL, LOX like–NE, Neutrophil elastase–MMP, Matrix metalloproteinase–SERPIN, serine protease inhibitor–TIMP, tissue inhibitors of metalloproteinases–EDP, Elastin derived peptides.

Collagens and elastin are the two most abundant and long-lasting components of the ECM. It's worth noting that in large elastic arteries, elastin is the predominant component compared to collagen (Duca et al., 2016). In the ECM, elastin, whose expression is undoubtedly regulated by the IGFRI-FOXO axis (Arabkhari et al., 2010; Conn et al., 1996; Shi et al., 2012; Zhao and Liu, 2021), is organized into EFs (Figure 1). Those fibers are a complex assembly of an elastin core surrounded by microfibrils arranged into concentric elastic lamellae in the wall of the large elastic arteries. Microfibrils are made up of fibrillins (1, 2, and 3), microfibril-associated glycoproteins (MAGPs), latent transforming growth factor beta binding protein (LTBP), fibulins, and emilin-1. Microfibril components, together with the cross-linking enzymes lysyl oxidase (LOX) and lysyl oxidase like 1 to 4 (LOXL1, -2, -3, -4), which establish intrafiber desmosin-isodesmosin bridges, facilitate the organization of tropoelastin monomers during elastin maturation process and are also involved in cell-elastic fiber interactions.

Elastin is an extremely durable protein with a half-life estimated to 70 years (Wahart et al., 2021) Elastin synthesis peaks at birth and is ended at the end of childhood. In adults, elastogenesis is almost non-existent. Therefore, from adolescence, a slow and progressive degradation of EFs is observed, affecting their functions. This process is central to vascular aging. Under physiological or pathological conditions inducing inflammation (Figure 1), elastases contribute to elastin degradation. Metalloproteinase-2 (MMP-2, gelatinase A), −9 (MMP-9, gelatinase B), −7 (MMP-7, matrilysin), and −12 (MMP-12, macrophage metalloelastase) are potent elastases. The secretion of leukocyte elastases and cathepsins S and G during inflammatory processes also leads to the increased degradation of elastin (Duca et al., 2016). Recently, a link has also been established between insulin resistance and the expression of neutrophil elastase (NE) or cathepsin S (Blaise et al., 2013; Lafarge et al., 2010; Lafarge et al., 2014; Mansuy-Aubert et al., 2013); this condition aggravates the fragmentation of elastin. The activity of proteases such as cathepsin S, NE, MMP-9 can be regulated by specific natural inhibitors such as serine protease inhibitors (serpins), cystatin C, TIMP-1, respectively (Kremastiotis et al., 2021; Lv et al., 2012; Mansuy-Aubert et al., 2013). These inhibitors, synthesized and secreted by numerous cells, are abundant in all body fluids and help prevent atherosclerotic, aneurysmal and high blood pressure events (Haves-Zburof et al., 2011; Kremastiotis et al., 2021; Lv et al., 2012; Rau et al., 2007; Romier et al., 2021). Nevertheless, the expression of these same inhibitors is dramatically reduced during metabolism syndrome, favoring then elastase activities (Mansuy-Aubert et al., 2013).

As a consequence, preserving the integrity of the ECM and, more particularly, protecting EFs from elastases is essential for maintaining arterial function (Duca et al., 2016). We therefore hypothesize that elastogenesis stimulation and elastolysis inhibition could reduce diabetes-induced elastic fiber aging such as we observed previously (Romier et al., 2021; Romier et al., 2018). As mentioned earlier, elastogenesis is a process that ceases during adolescence. Elastogenesis can only be induced in adults through the introduction of exogenous molecules. Currently, minoxidil treatment is the only one described as capable of inducing the neo-synthesis of functional elastic fibers in human (Knutsen et al., 2022) and in several mice models including aged mice (Coquand-Gandit et al., 2017; Fhayli et al., 2019) or elastin deficient mice (Knutsen et al., 2018; Knutsen et al., 2022). Minoxidil is described as a potassium channel opener, suggesting that the de novo-synthesis of elastic fibers could be under the control of potassium channels. However, minoxidil does have significant side effects. Therefore, it is crucial to test other antihypertensive agents. Many antihypertensive drugs targeting, for example, angiotensin, also exert a significant metabolic influence (Gillespie et al., 2005; Putnam et al., 2012; Sowers). This is why we prioritized beta-blocking molecules and tested nebivolol, which would have negligible metabolic effects (Deedwania et al., 2013; Toblli et al., 2010) while also potentially impacting potassium channels (Altunkaynak-Camca, 2020; Illiano et al., 1992; Tinker et al., 2014). Furthermore, nebivolol has been described as a cardiac fibrosis inhibitor, thus demonstrating a significant impact on the ECM of the heart and suggesting potentially, a role on vascular ECM remodeling (Lin et al., 2013; Toblli et al., 2010; Ye et al., 2013).

In the present work, we assessed the ability of two antihypertensive molecules, nebivolol (a β-blocker) and minoxidil, to change the elastogenesis–elastolysis balance toward a net neosynthesis of EFs, in a model of diabetic mice that no longer expressed the leptin receptor (db/db). Minoxidil is an established K + ATP channel opener shown to induce neo-elastogenesis in aged or elastin-deficient models. However, its known cardiovascular side effects limit its long-term use in clinical settings. Nebivolol, a β1-blocker with β3-agonist and nitric oxide (NO)–mediated vasodilatory properties, has demonstrated antioxidant effects and endothelial protective actions. These features are particularly relevant in the diabetic context, which is characterized by chronic oxidative stress and endothelial dysfunction. Both agents have shown beneficial effects on vascular remodeling and stiffness, which are hallmark features of diabetic vasculopathy. Therefore, in the present work, we assessed the ability of two antihypertensive molecules, nebivolol (a β-blocker) and minoxidil, to the development of effective therapeutic strategies aiming at limiting the vascular complications of diabetes in this animal model.

Finally, we identified the involved molecular targets of these compounds as a preliminary step to the development of effective therapeutic strategies aiming at limiting the vascular complications of diabetes in this animal model.

## Methods

### Experimental models

Detailed methods for cell culture are described in the Supplementary Methods section. *Animal models*–All mouse procedures are conformed to the Guide for Care and Use of Laboratory Animals of the US National Institutes of Health. The study respected European and French legislation on good practices in animal experimentation and was approved by the Animal Subjects Committee of Champagne Ardenne (CEEA-RCA-56). Male db/db mice (C57BL6/J as genetic background) are 6 months old in order to test the treatments on a severe diabetic model presenting significant cardiovascular alterations in accordance with the literature (Daniels Gatward et al., 2021; Gomes et al., 2021; Gomes et al., 2022). Metabolic parameters (food and water intakes, HbA1c, Glycemia, and body weight) were described in Table 1. We used 6-month-old non-diabetic control mice (C56BL6/J, n = 10) as a reference group. Mice were purchased from Janvier (Le Genest-Saint-Isle, France) All mice had *ad libitum* access to a standard diet (AIN-93 M rodent diet, Special Diet Service, United Kingdom) and water during the experimental period. The drinking water (renewed daily) of db/db mice contained (or not) minoxidil (20 mg/kg/day) or nebivolol (20 mg/kg/day) for 8 weeks. The concentrations and treatment times were adapted to previous data from the literature using minoxidil as a therapeutic molecule for ECM alterations.

**TABLE 1:** Metabolism parameters.

Parameters	Control C57Bl6J (n = 10)	db/db untreated (n = 10)	db/db treated with minoxidil (n = 10)	db/db treated With nébivolol (n = 10)
Food intake (g/day/animal)	3.56 ± 0.64	5.69 ± 0.52^c^	5.58 ± 0.54^c^	4.99 ± 0.45^c^
Water intake (ml/day/animal)	3.84 ± 0.86	7.57 ± 1.11^c^	6.81 ± 0.94^c^	7.21 ± 0.91^c^
HbA1c (%)	4.76 ± 0.41	8.95 ± 1.39^c^	7.46 ± 0.81^c^	8.66 ± 0.92^c^
Glycemia (mg/dL)	171.4 ± 31.3	426.4 ± 65.3^c^	372.6 ± 100.1^c^	429.4 ± 94.64^c^
Body weight (g)	26.45 ± 1.31	35.93 ± 4.12^c^	39.0 ± 3.23^c^	38.75 ± 3.68^c^

The results are the mean ± SEM., Statistically significant differences: c (*versus* control), p < 0.05.

### Physiological parameters

*Blood pressure assay*–Preconditioning was performed within 7 days before the final measurement of blood pressure. For this purpose, the animals were acclimated to contention and to the pressure of the sphygmomanometer on their tails. The day of the final measure, five “empty” measurements were carried out within a 60 s interval. The final measurement was the average of five successive measurements. If the systolic and/or diastolic pressures of the mice (aged C57Bl/6 or treated or untreated young db/db) were statistically superior to the pressure of the control mice, we considered the mice to be hypertensive. *Pulse wave velocity*–Pulse wave velocity measurement was performed non-invasively using the ultrasound Doppler Flow Velocity System (Indus Instruments, Webster, United States) on anesthetized mice. The heart rate and ejection time between the femoral aorta and the aortic arch were measured, making possible to determine the speed of the pulse wave (pulse wave velocity) expressed in m/s. The latter was calculated by dividing the distance between the two Doppler probes by the ejection time between the two regions of the aorta. *High frequency ultrasound analysis* was performed using Vevo imaging systems (Fujifilm VisualSonics, Toronto, Canada) and under light isoflurane anesthesia (1.5%–2% in oxygen), with acquisition completed within a maximum of 10 min per animal, in accordance with ethical guidelines and to minimize cardiovascular depression in db/db mice. Heart rate was monitored to remain within the recommended physiological range (400–600 bpm), and animals were maintained on a heated platform to prevent hypothermia. As isoflurane can affect systolic parameters by reducing preload and contractility, we standardized all procedures to limit variability across animals and ensure comparable conditions across groups. At the level of the aortic arch, the diameter of the vessel was measured during the cardiac cycle (systole, Ds-diastole, Dd), as well as the pulse wave velocity (PWV) and the thickness of the tunica intima-media (h). Distensibility factor (DC) and Young’s modulus (E) were derived by Bramwell, (1922) equation and Moens-Korteweg equation, respectively. From the conclusions of Brands et al. (1999), the local variation of the pressure during the cardiac cycle (DP) and the compliance (CC) can then be deduced (Vanalderwiert et al., 2024a).

### Imaging methods

Detailed methods for histology and Immunofluorescent staining are described in the Supplementary Methods section. *Atomic force microscopy (AFM)* – Frozen 10 μm-thick aorta cross-sections were thawed in Krebs–Henseleit solution. After locating the zones of interest, analysis was performed using AFM (Bioscope Catalyst, Bruker, Billerica, United States, driven by Nanoscope Analysis 1.8 software) coupled to a Nikon Eclipse Ti inverted microscope (Nikon, Tokyo, Japan). The young modulus at each point of the EFs or of the interfiber spaces was calculated using a value of the Poisson ratio of 0.5 for our samples considered incompressible. For each condition, at least 5,000 force curves were treated to obtain the mean YM values for the EFs and the interfiber spaces. The analyses were performed at three different locations in each cross-section, for a total of nine cross-sections obtained from three different mice.

### Biochemical methods

Detailed methods for immunoblotting, qPCR are described in the Supplementary Methods section. *Extracellular elastin quantification* was performed by ELISA test. After 48 h of incubation (with potassium channel modulators), MOVAS cell cultures (no impermeabilized) were incubated at 37 °C (2 h) with anti-Elastin antibody (BA41/1000, Sigma), then secondary antibody coupled to HRP (1 h, 37 °C). 3,3′,5,5′-tetramethybenzidin (TMB) was added in each well (5 min of incubation), followed by sulfuric acid (0.3M, for 10 min) to stop the reaction. Absorbance was read at 450 nM. Plasma marker assays - Peripheral blood of mice was collected in heparinized tubes by retroorbital puncture, and plasma were stored at −80 °C. *Evaluations of desmosine and elastin-derived peptides (EDP)* concentrations were performed using commercially available kits (Cusabio Biotech Product, Houston TX, United States and Biocolor, County Antrim, United Kingdom, respectively). The NE and cathepsin activity assay commercialized by Abcam (Cambridge, United Kingdom) were used to measure NE and cathepsin S activities, respectively, according to the method described by Romier et al. (Sarafidis et al., 2017). *Tissular total collagen quantity* was determined using a QuickZyme Total collagen kit (QuickZyme Biosciences, Leiden, the Netherlands). Crosslink and elastin quantification- Protein analysis was performed as described by Romier et al. (Olawi et al., 2019). Briefly, for *collagen crosslink analysis* samples were reduced by sodium borohydride (Sigma-Aldrich, Germany) (25 mg NaBH4/ml in 0.05 M NaH2PO4/0.15 M NaCl pH 7.4, 1 h on ice, 1.5 h at room temperature) and digested with high purity bacterial collagenase (C0773; Sigma, Germany; 50 U/mL, 37 °C, 12 h). The soluble fractions containing collagen cross-links were hydrolyzed in 6 N HCl at 110 °C for 24 h. The hydrolysates were precleared by solid phase extraction. Dried eluates were analyzed with an amino acid analyzer (Biochrom 30, Biochrom, Great Britain). The nomenclature of the crosslinks used in the article refers to the reduced variants of crosslinks. The collagen content was analyzed in an aliquot of hydrolyzed samples of the collagenase soluble fraction prior to preclearence and calculated based on a content of 14 mg hydroxyproline in 100 mg collagen. For *protein and elastin crosslinks analysis*, samples were digested with bacterial collagenase. The soluble fraction containing collagen was subjected to hydrolysis and amino acid analysis. The residual fraction was extracted by hot alkali (0.1 N NaOH, 95 °C, 45 min). The supernatant containing non-collagenous/non-elastin proteins and the insoluble residue containing insoluble elastin were subjected to hydrolysis and amino acid analysis. The content of elastin crosslinks was analyzed in an aliquot of the NaOH-insoluble fraction containing elastin after CF-11 preclearence by amino acid analysis.

### Statistical analyses

Data were prospectively collected and analyzed using StatView 5.0 software for Windows (SAS Institute, Berkley, CA). Results are presented as either boxplots (with minimum and maximum values) or bar graphs showing mean ± SEM, to reflect the precision of the estimated means in a multi-replicate experimental design. Group comparisons were performed using non-parametric ANOVA (Kruskal–Wallis test), followed by pairwise Wilcoxon–Mann–Whitney tests with Bonferroni–Dunn correction for multiple comparisons. All reported p-values, including those displayed in figures and tables, are adjusted (corrected) p-values. These are explicitly indicated in the figure legends. Each analysis was based on at least three independent experiments, each including a minimum of five animals per group. A corrected p-value <0.05 was considered statistically significant. In all figures and tables, “*” denotes a corrected p-value <0.05 between untreated db/db mice and those treated with minoxidil or nebivolol, while “c” denotes a corrected p-value <0.05 between untreated db/db mice and C57Bl6 control mice.

## Results

### Minoxidil treatment reduces aorta stiffening in diabetic mice

In order to evaluate its efficiency in limiting the premature aging of the aorta wall observed in db/db mice, we administrated minoxidil to diabetic mice for 8 weeks in conditions comparable to those used to stimulate neosynthesis/protection of arterial EFs in young rats and aged mice (Coquand-Gandit et al., 2017; Fhayli et al., 2019).

**FIGURE 2:**
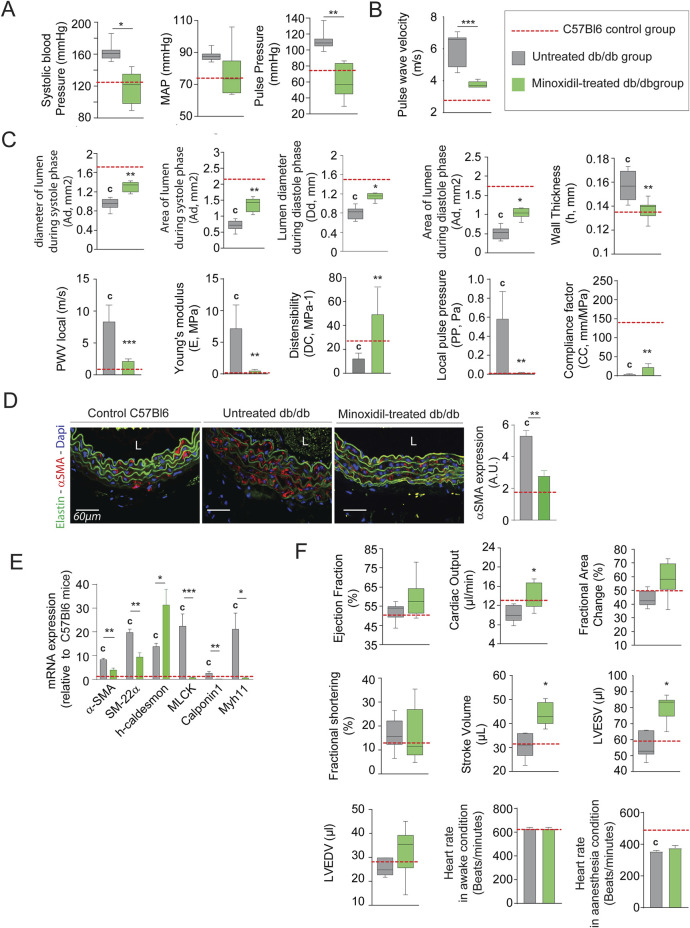
Minoxidil treatment improves aortic and cardiac function in db/db mice. **(A)** Systolic, diastolic, mean arterial (MAP), and pulse blood pressures (n = 10/group); **(B)** pulse wave velocity (PWV) in the thoracic aorta (n = 10/group); **(C)** aortic ultrasound-derived functional parameters including systolic (Ds) and diastolic (Dd) diameters, wall thickness (h), systolic (As) area of diastole (Ad) lumen areas, local distensibility coefficient (DC), local compliance coefficient (CC), and Young’s modulus **(E)**, calculated using standard hemodynamic equations (see Methods) (n = 10/group) (n = 10/group); **(D)** representative aortic cross-sections stained for α-SMA (red), nuclei (DAPI, blue), and elastin autofluorescence (green); quantification of α-SMA signal intensity (ImageJ) (n = 10/group); **(E)** relative aortic mRNA expression of smooth muscle markers: α-SMA, SM22α, h-Caldesmon, MCLK, Calponin-1, and Myh11 (n = 10/group); **(F)** cardiac ultrasound parameters including ejection fraction, fractional area change, stroke volume, LVEDV, LVESV, and heart rate in anesthetized and non-anesthetized conditions (n = 10/group). Data are expressed as mean ± SEM from at least three independent experiments. n = 10 mice per group (except F: n = 5). Green bars: minoxidil-treated db/db mice; grey bars: untreated db/db mice; red dashed line: mean value from C57Bl/6 control mice (n = 10, shown as reference only). Statistical analysis: Kruskal–Wallis test followed by Wilcoxon–Mann–Whitney *post hoc* test with Bonferroni correction. All shown p-values are corrected.

**FIGURE 3:**
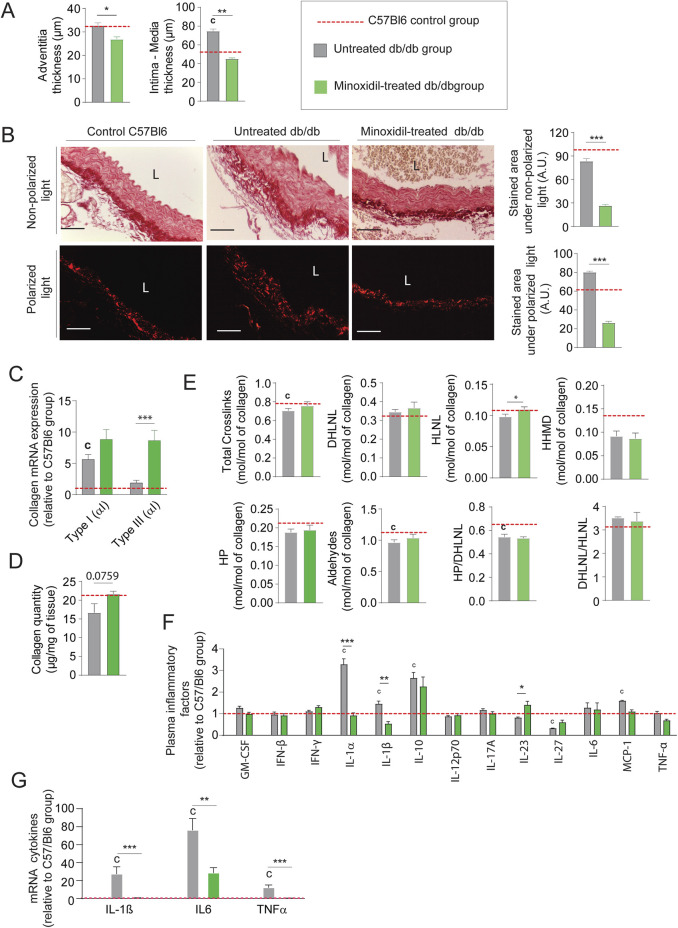
Minoxidil reduces collagen accumulation and inflammation in db/db mice. **(A)** Aortic intima-media and adventitia thicknesses (n = 10/group); **(B)** representative aortic cross-sections stained with picrosirius red (n = 10/group), observed under non-polarized light (total collagen) and polarized light (type I and III collagen); quantification of collagen area performed using ImageJ. L = lumen. Scale bars: 120 μm; **(C)** aortic mRNA expression of collagen types I and III (Col1a1, Col3a1) (n = 10/group); **(D)** total collagen content, (n = 5/group); **(E)** collagen cross-linking parameters (n = 5/group), including hydroxypyridinoline (HP), dihydroxylysinonorleucine (DHLNL), hydroxylysinonorleucine (HLNL), and histidinohydroxymerodesmosine (HHMD); total aldehydes calculated as: 2×HP + DHLNL + HLNL +2×HHMD; **(F)** aortic mRNA levels of inflammatory markers (e.g., IL-6, IL-1β); (n = 5/group); **(G)** plasma levels of inflammatory cytokines measured by ELISA (n = 5/group). Data are presented as mean ± SEM from at least three independent experiments. Green bars: minoxidil-treated db/db mice; grey bars: untreated db/db mice; red dashed line: C57Bl/6 control group (n = 5–10, shown for reference only). Statistical analysis: Kruskal–Wallis test followed by Wilcoxon–Mann–Whitney *post hoc* test with Bonferroni correction. All displayed p-values are corrected. *p < 0.05 vs. untreated db/db; c: p < 0.05 vs. C57Bl/6 control mice.

Minoxidil-reduced systolic and pulse blood pressures (not mean arterial pressure (MAP)), as well as aortic pulse wave velocity or compliance and distensibility measured using the high ultrasound method, were indicative of arterial stiffness (Figures 2A–C, respectively). This was associated with a decrease in expression of the smooth muscle cells (SMC) contraction markers α-SMA, SM-22α, MLCK (Myosin light-chain kinase), Calponin and MYH11 (smooth muscle myosin heavy chain) (Figures 2D,E; Supplementary Figure 1B). These data demonstrated the antihypertensive and anti-aortic stiffening efficacy of minoxidil in diabetic animals but did not affect glycemic parameters (Table 1). Figure 2C suggests that minoxidil administration might be sufficient to restore an almost normal aortic function (observed by the dashed red line obtained with C57Bl6 mice (n = 10)). Minoxidil also induced aorta wall remodeling, leading to decreased intima-media and adventitia thicknesses (Figure 3A and Supplementary Figure 1B). This is possibly due to a decrease in total wall collagen content, particularly type III and I collagen as we observed by red picrosirius staining (Figure 3B). The increase of the mRNA expression of these collagens (Figure 3C) together with the absence of increased total collagen (Figure 3D) contents and crosslinks (Figure 3E) suggest the degradation of neosynthesized collagens. Because chronic inflammation, through the production of cytokines such as TNF-α and IL-6, can promote tissue fibrosis, we sought to determine whether minoxidil treatment affects inflammatory factors that could explain changes in collagen expression. We found that minoxidil significantly reduces the production of pro-fibrotic cytokines both at the plasma level (Figure 3F) and in the aorta of db/db mice (Figure 3G).

### Minoxidil treatment promotes elastogenesis while reducing elastolysis, in diabetic mice

**FIGURE 4:**
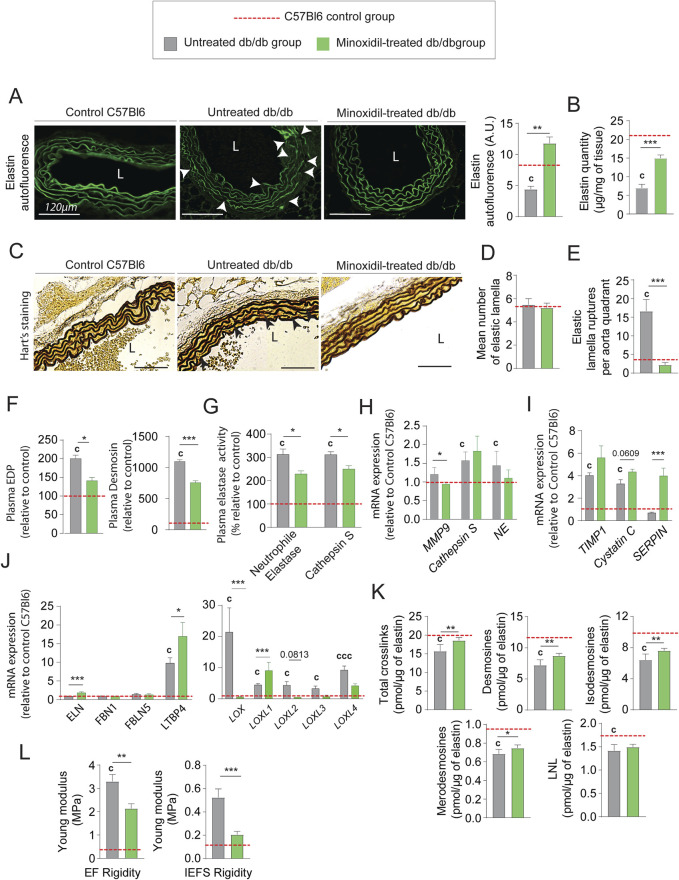
Minoxidil improves elastin content and elastic fiber integrity in the aorta of db/db mice. **(A)** Elastin autofluorescence in thoracic aorta cross-sections (L = lumen, scale bars: 120 µm) and quantification using ImageJ (n = 10/group); **(B)** quantification of insoluble elastin content (n = 10/group); **(C)** representative images of Hart’s staining for elastic fibers (EFs) (scale bars: 120 µm) (n = 10/group); **(D)** number of elastic lamellae (EL) per section (n = 10/group); **(E)** quantification of lamellar ruptures per aortic quadrant using elastin autofluorescence (n = 10/group); **(F)** plasma levels of elastin-derived peptides (EDP) and desmosine (n = 10/group); **(G)** plasma activities of neutrophil elastase (NE) and cathepsin S (n = 10/group); **(H)** aortic mRNA levels of elastolytic markers: MMP-9, NE, and cathepsin S (n = 10/group); **(I)** aortic mRNA expression of natural elastase inhibitors: TIMP1, cystatin C, and SERPIN (n = 10/group); **(J)** aortic mRNA expression of elastogenesis-related genes: elastin (ELN), fibrillin-1 (FBN1), fibulin-5 (FBLN5), LTBP4, LOX, and LOXL1–4 (n = 10/group); **(K)** aortic content of elastin-specific cross-links (desmosines and isodesmosines, n = 10/group); **(L)** atomic force microscopy (AFM)-based quantification of elastic lamellae (EL) and inter-elastic lamellae space (IELS) stiffness (n = 10/group). Data are shown as mean ± SEM from at least three independent experiments. Green bars: minoxidil-treated db/db mice (n = 10); grey bars: untreated db/db mice (n = 10); red dashed line: reference values from C57Bl/6 mice (n = 10, shown for visual comparison only). Statistical analysis: Kruskal–Wallis test followed by Wilcoxon–Mann–Whitney *post hoc* test with Bonferroni correction. All displayed p-values are corrected. *p < 0.05 vs. untreated db/db; c: p < 0.05 vs. C57Bl/6 control mice.

Elastin autofluorescence level (Figure 4A) and elastin quantity (Figure 4B; Supplementary Figure 2A) are increased, suggesting an increase in elastic fiber content and/or a decreased elastic fiber degradation after minoxidil treatment, as shown by elastin autofluorescence (Figure 4A) and Hart’s staining (Figure 4C). Indeed, while the number of elastic lamellae remained unchanged by minoxidil treatment (Figure 4D). Likewise, the number of elastic lamellae ruptures (Figure 4E) is decreased by the treatment reflecting a restructuring of lamellae. This reduced fragmentation of the elastic networks is confirmed by a significant decrease in plasmatic elastin-derived peptides (EDP) and desmosine levels (Figure 4F). Regarding elastolysis, the activities of cathepsin S and NE were reduced by the treatment (Figure 4G), while the tissue proteinase mRNA levels were unaffected by minoxidil (Figure 4H). The minoxidil-induced decrease in elastolysis in the aorta was amplified by the stimulatory effect of the treatment on the increased tissular expression of natural proteinase inhibitor, such as Serine peptidase inhibitor (SERPIN) (Figure 4I). The fact that minoxidil treatment stimulated aortic elastogenesis is further suggested by elevated mRNAs levels for elastin, LTBP4, and LOXL1 (Figure 4J). Increased and efficient elastogenesis was demonstrated by the increase of cross-links following the treatment (Figure 4K), as the occurrence of these cross-links is an indicator of the maturity and functionality of EFs. Inhibition of elastolysis and induction of elastogenesis by minoxidil improved elastic fiber content and function, as indicated by the decrease of elastic lamellae and interlamellar space stiffnesses in diabetic mice (Figure 4L). Taken together, these observations show that minoxidil limits premature aging of the aortic wall in diabetic mice and partially restores aorta function. Nevertheless, the cardiac parameter data (Figure 2F) show that, following the administration of minoxidil, cardiac stroke volume and left ventricle end-diastolic volume increased in db/db mice. The data highlights the fact that problems associated with alterations in left ventricular (LV) function can eventually impact aortic function and structure over time. This could compromise the anti-vascular aging effects of the medications used in our study and others (Coquand-Gandit et al., 2017; Knutsen et al., 2018; Knutsen et al., 2022).

### Chronic nebivolol treatment limits the progression of aortic stiffness induced by diabetes

**FIGURE 5:**
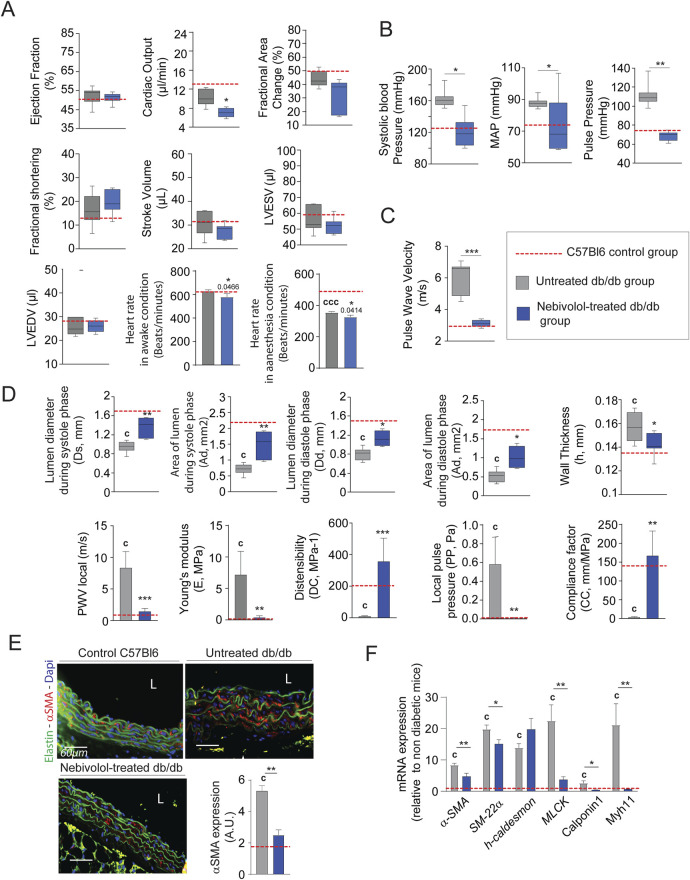
Nebivolol improves cardiovascular function and aortic elasticity in db/db mice. **(A)** Left ventricular function assessed by high-frequency ultrasound (n = 10/group), including ejection fraction, fractional area change, fractional shortening, stroke volume, LVEDV, and LVESV; heart rate measured under anesthetized and conscious conditions; **(B)** systolic, diastolic, mean arterial (MAP), and pulse blood pressures (n = 10/group); **(C)** pulse wave velocity (PWV) in the thoracic aorta (n = 10/group); **(D)** aortic ultrasound-derived functional parameters (n = 10/group): systolic (Ds) and diastolic (Dd) diameters, wall thickness (h), lumen surface areas during systole (As) area of diastole (Ad), calculated metrics include distensibility coefficient (DC), Young’s modulus **(E)**, pulse pressure (PP), and compliance coefficient (CC), as detailed in the Methods section; **(E)** representative cross-sections of thoracic aorta stained for α-smooth muscle actin (α-SMA, red), nuclei (DAPI, blue), and elastin autofluorescence (green); scale bars: 60 μm; ImageJ-based quantification of α-SMA signal (n = 10/group) **(F)** aortic mRNA expression of smooth muscle cell markers: α-SMA, SM22α, h-Caldesmon, MCLK, Calponin-1, and Myh11 (n = 10/group). Data are expressed as mean ± SEM from at least three independent experiments. Blue bars: nebivolol-treated db/db mice (n = 10); grey bars: untreated db/db mice (n = 10); red dashed line: reference values from C57Bl/6 mice (n = 10, shown for comparison only). Statistical analysis: Kruskal–Wallis test followed by Wilcoxon–Mann–Whitney *post hoc* test with Bonferroni correction. All displayed p-values are corrected. *p < 0.05 vs. untreated db/db; c: p < 0.05 vs. C57Bl/6 control mice.

Minoxidil chronic treatment can compensate for elastolysis induced by diabetes. However, this treatment has several side effects on cardiac functions, which limit its use (see Figure 2F) (Hanton et al., 2008). For this reason, we sought to test other antihypertensive molecules that could have similar beneficial effects without these harmful side effects. Among antihypertensive molecules, pharmacological blockade of the β1-adrenergic receptor can extend the life span of mice and flies, independently of body weight or metabolic syndrome (Spindler et al., 2013). Therefore, we chose to evaluate the effect of nebivolol, which has been described as a β-blocker reducing heart rate (Figure 5A), and a potent vasodilator activating β2 adrenergic receptors in endothelial cells and SMCs (Olawi et al., 2019; Toblli et al., 2010). Figure 5B shows that treatment with nebivolol for 8 weeks is sufficient to reduce systolic, mean, and pulse arterial pressures. We also observe nebivolol-induced decreases in aortic pulse wave velocity (Figure 5C), which favors the compliance of thoracic aorta and decreases Young’s modulus (Figure 5D), and expression of vasoconstriction factors such as SM-22α, α-SMA, MLCK, Calponin and Myh11 (Figures 5E,F). Interestingly, Figures 5B–D demonstrate that nebivolol restores aortic functions, at levels observed in young, non-diabetic mice.

### Nebivolol treatment protects EFs and stimulates neosynthesis in diabetic mice

**FIGURE 6:**
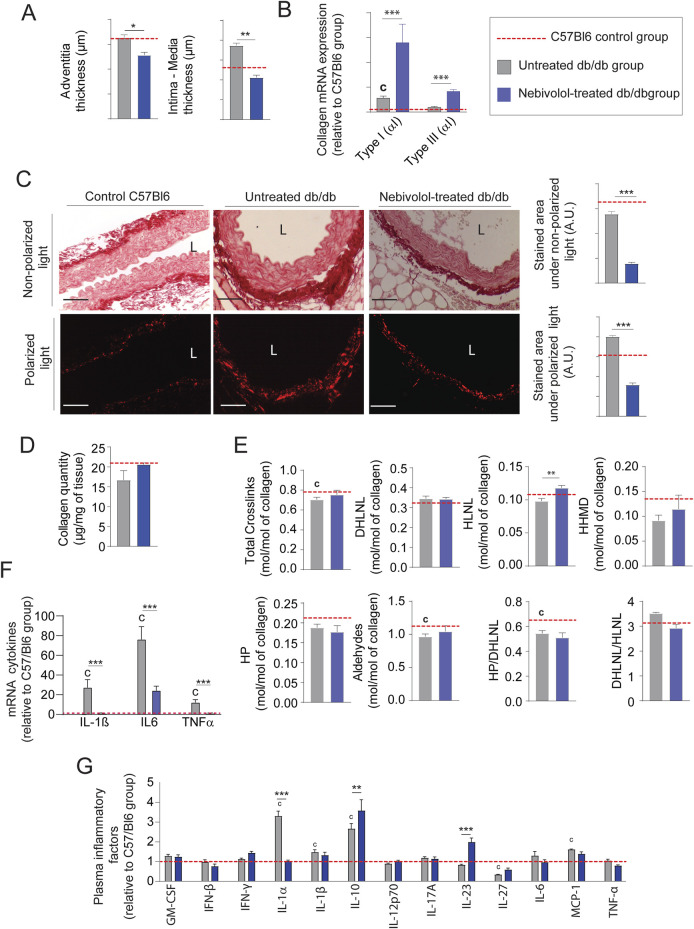
Nebivolol reduces collagen accumulation and inflammation in db/db mice. **(A)** Aortic intima-media and adventitia thicknesses (n = 10/group); **(B)** representative aortic cross-sections stained with picrosirius red (n = 10/group), imaged under non-polarized light (total collagen) and polarized light (type I and III collagen); quantification performed using ImageJ; L = lumen; scale bars: 120 μm; **(C)** aortic mRNA expression of collagen type I (Col1a1) and type III (Col3a1) (n = 10/group); **(D)** total collagen content (n = 5/group); **(E)** quantification of collagen cross-linking (n = 5/group), including hydroxypyridinoline (HP), dihydroxylysinonorleucine (DHLNL), hydroxylysinonorleucine (HLNL), and histidinohydroxymerodesmosine (HHMD); total aldehydes calculated as: 2×HP + DHLNL + HLNL +2×HHMD; **(F)** aortic mRNA levels of inflammatory markers (n = 5/group); **(G)** plasma concentrations of inflammatory cytokines measured by ELISA (n = 5/group). Data are presented as mean ± SEM from at least three independent experiments. Green bars: nebivolol-treated db/db mice; grey bars: untreated db/db mice; red dashed line: C57Bl/6 control group (n = 5–10, shown for reference only). Statistical analysis: Kruskal–Wallis test followed by Wilcoxon–Mann–Whitney *post hoc* test with Bonferroni correction. All displayed p-values are corrected. *p < 0.05 vs. untreated db/db; c: p < 0.05 vs. C57Bl/6 control mice.

**FIGURE 7:**
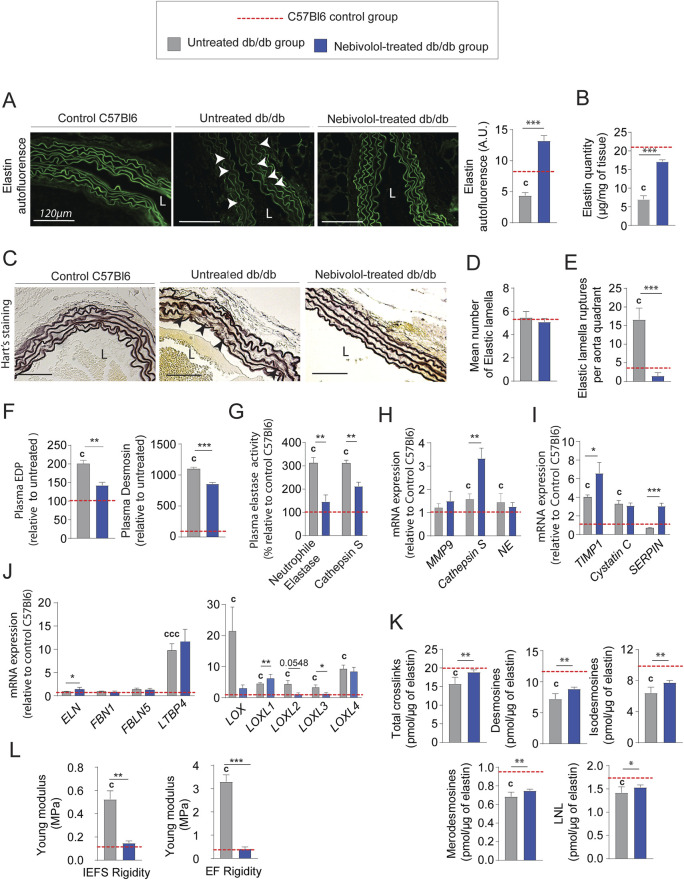
Nebivolol treatment improves elastin integrity and reduces aortic aging features in db/db mice. **(A)** Elastin autofluorescence in thoracic aorta cross-sections and ImageJ-based quantification (n = 10/group); L = lumen; scale bars: 120 μm; **(B)** quantification of insoluble elastin content (n = 10/group); **(C)** representative Hart’s-stained sections showing elastic fibers (EFs); scale bars: 120 µm (n = 10/group); **(D)** number of elastic lamellae (EL) per aortic section (n = 10/group); **(E)** number of lamella ruptures per quadrant determined by autofluorescence (n = 10/group); **(F)** plasma levels of elastin-derived peptides (EDPs) and desmosine (n = 10/group); **(G)** plasma enzymatic activities of neutrophil elastase (NE) and cathepsin S (n = 10/group); **(H)** aortic mRNA expression of elastolytic markers: MMP-9, NE, and cathepsin S (n = 10/group); **(I)** aortic mRNA expression of natural elastase inhibitors: TIMP1, cystatin C, and SERPIN (n = 10/group); **(J)** aortic mRNA levels of elastogenesis-related genes: elastin (ELN), FBN1, FBLN5, LTBP4, LOX, and LOXL1–4 (n = 10/group); **(K)** desmosine and isodesmosine cross-link quantification from thoracic aorta (n = 5/group); **(L)** stiffness of elastic lamellae (EL) and inter-elastic lamellae space (IELS) measured by atomic force microscopy (AFM) (n = 10/group). Data are presented as mean ± SEM from at least three independent experiments. Blue bars: nebivolol-treated db/db mice; grey bars: untreated db/db mice; red dashed line: C57Bl/6 control values (n = 10, shown for reference only). Statistical analysis: Kruskal–Wallis test followed by Wilcoxon–Mann–Whitney *post hoc* test with Bonferroni correction. All displayed p-values are corrected. p < 0.05 vs. untreated db/db; c: p < 0.05 vs. C57Bl/6 control mice.

From an anatomical point of view, treatment with nebivolol also significantly reduced adventitia and media-intima thicknesses (Figure 6A and Supplementary Figure 1B). Importantly, collagen modifications cannot explain by itself thicknesses reduction after treatment. Indeed, we noticed that the mRNA expression of types I and III were significantly increased (Figure 6B) while collagen staining by red picrosirius, in the aortic adventitia was reduced (Figure 6C). Moreover, total collagen level (Figure 6D) and crosslinks quantities (Figure 6E) were comparable to those observed in untreated db/db mice. Similar to minoxidil, nebivolol treatment induces a decrease in pro-fibrotic inflammatory factors in the aorta of diabetic mice (Figure 6F) as well as in their plasma (Figure 6G) compared to untreated db/db mice. Using Western blotting (Supplementary Figure 2), elastin autofluorescence (Figure 7A) and extraction of insoluble elastin (Figure 7B), we demonstrated an increase of elastin quantity after nebivolol treatment. Using Hart’s staining (Figure 7C) or by elastin autofluorescence (Figure 7A), we observed that, although the number of elastic lamellae was unchanged in the media (Figure 7D), their integrity was improved by nebivolol treatment. This was evidenced by the dramatic decrease in elastic lamellae ruptures (Figure 7E), and the decreased plasma levels of EDP and desmosines (Figure 7F). In parallel, nebivolol treatment decreased plasma cathepsin S and NE activities (Figure 7G), while, surprisingly, the expression of cathepsin S within the aorta (Figure 7H) was increased. The expression of the natural inhibitors of MMP9 and NE elastases, say TIMP1 and SERPIN, was significantly increased by nebivolol treatment (Figure 7I). Nebivolol treatment also induced the expression of elastin and LOXL1 and decreased LOXL3 expression (Figure 7J), suggesting a positive effect on elastogenesis. Figure 7K shows that this neosynthesis of elastin (Supplementary Figure 2) was associated with an increase of all crosslink classes, suggesting the formation of mature and functional elastin. The consequence of the treatment was a spectacular drop in aortic rigidity (by 5–10 times), as measured by AFM (Figure 7L). Altogether, these findings suggest that chronic treatment with nebivolol can limit the EFs premature aging, with fewer cardiovascular side effects.

### Minoxidil and nebivolol treatments stimulate elastogenesis and limit elastolysis in cultured vascular SMCs

**FIGURE 8:**
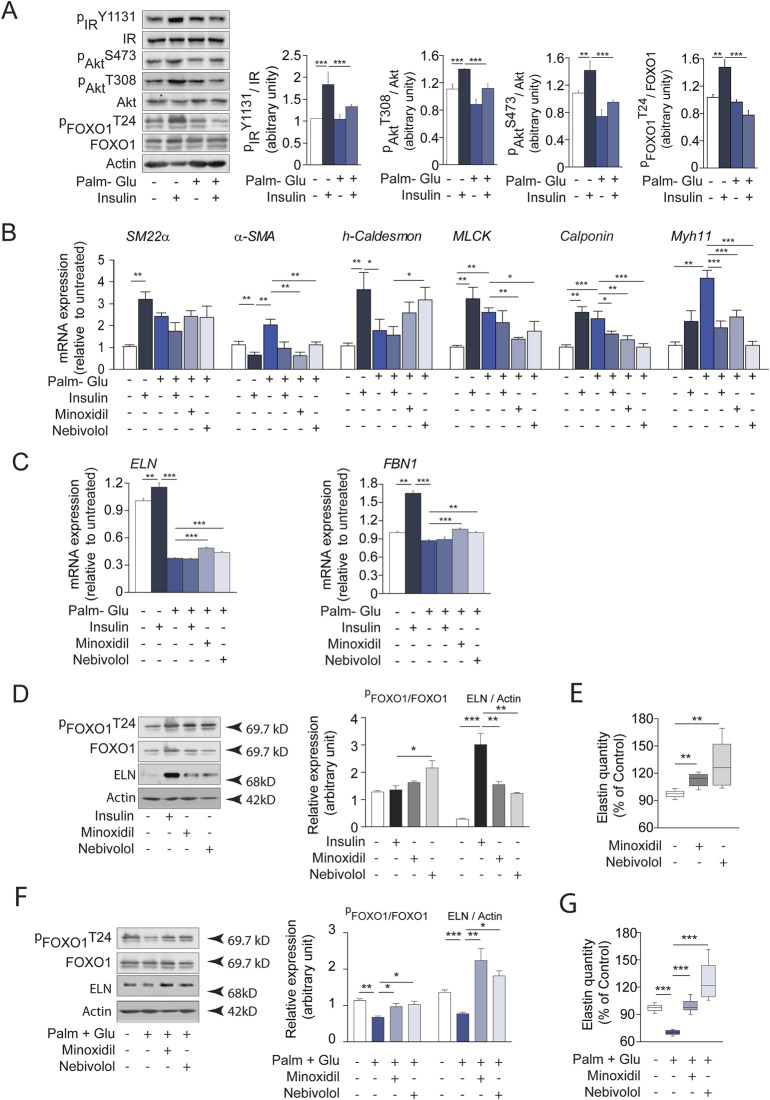
Insulin, minoxidil, and nebivolol modulate elastogenesis and FOXO1 signaling in cultured insulin-resistant vascular smooth muscle cells (SMCs). **(A)** Western blots showing native and phosphorylated forms of insulin receptor (IR), Akt, and FOXO1 in MOVAS cells incubated with 20 mM glucose and 0.5 mM palmitate (Glu + Palm, 48 h), and stimulated or not with 100 nM insulin for 15 min; right panels: semi-quantification by ImageJ (n = 3/group); **(B)** mRNA expression of smooth muscle contractile markers: α-SMA, SM22α, h-Caldesmon, MLCK, Calponin, and Myh11 (n = 3/group); **(C)** mRNA expression of elastogenesis markers: elastin (ELN) and fibrillin-1 (FBN1) (n = 3/group); **(D)** Western blot analysis of FOXO1 (native and phosphorylated), ELN, and actin after 48 h incubation with minoxidil or nebivolol, or 15 min insulin stimulation; right panel: Band intensities were quantified by densitometry using ImageJ (n = 3/group); **(E)** Quantification of extracellular elastin secretion (ELISA, 450 nm absorbance) in control conditions (n = 8/group); **(F)** Western blots analysis of FOXO1 and ELN in insulin-resistant conditions (Glu + Palm) with or without 48 h minoxidil or nebivolol; right panel: Band intensities were quantified by densitometry using ImageJ (n = 3/group); **(G)** Extracellular elastin secretion in Glu + Palm–treated MOVAS cells incubated with or without minoxidil or nebivolol (n = 8/group). Data are presented as mean ± SEM from at least three independent experiments. Statistical analysis: Kruskal–Wallis test followed by Wilcoxon–Mann–Whitney *post hoc* test with Bonferroni correction. All displayed p-values are corrected. p < 0.05 vs. untreated.

**FIGURE 9:**
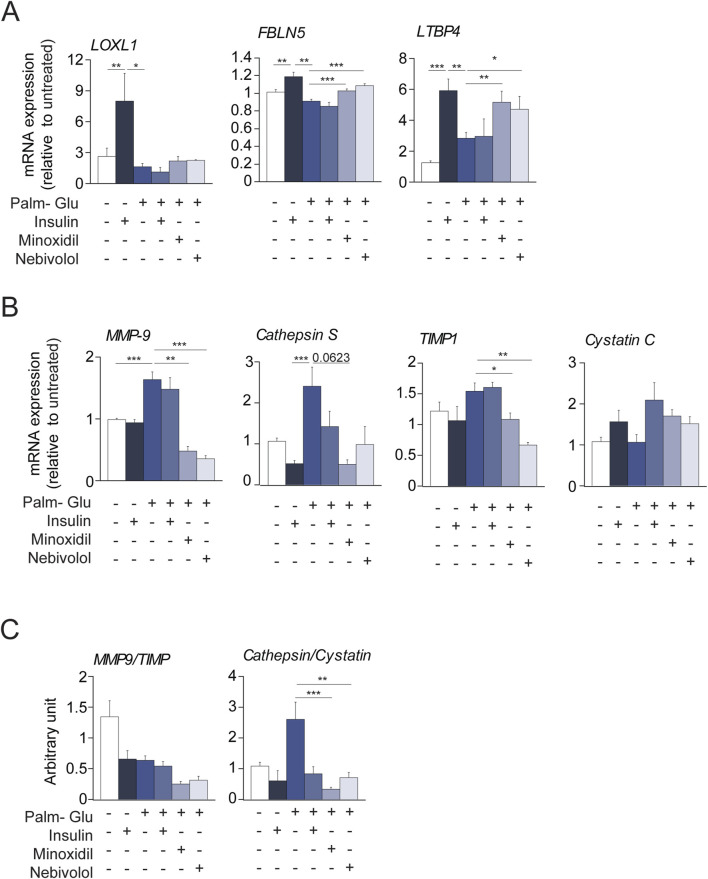
Minoxidil and nebivolol regulate elastogenesis and elastolysis gene expression in insulin-resistant vascular SMCs. **(A)** mRNA levels of FBLN5, LTBP4, and LOXL1 (n = 3/group); **(B)** expression of MMP-9, cathepsin S, and their respective inhibitors TIMP1 and cystatin C (n = 3/group); **(C)** proteinase/inhibitor ratios: MMP-9/TIMP1 and cathepsin S/cystatin C (n = 3/group). Data are presented as mean ± SEM from at least three independent experiments. Statistical analysis: Kruskal–Wallis test followed by Wilcoxon–Mann–Whitney *post hoc* test with Bonferroni correction. All displayed p-values are corrected. p < 0.05 vs. untreated.

As described in the literature (Inyard et al., 2009; Standley et al., 1991), diabetes promotes *in vivo* vascular smooth muscle contraction processes and the expression of elastolysis markers. In contrast, the use of the antihypertensive molecules minoxidil or nebivolol in diabetic animals induced a decrease in the markers of SMC contraction and an increase in the markers of elastogenesis. In this context, we aimed to focus on the effects of the treatments, Minoxidil and Nebivolol, on the behavior of insulin-resistant smooth muscle cells (SMCs). For this purpose, SMCs were pre-incubated with a medium enriched in glucose-palmitate. To maintain consistency between *in vivo* models (male db/db mice from the C57/Bl6J strain) and *in vitro* conditions, we opted to utilize the MOVAS cell line, derived from SMCs of male C57Bl6J mice. Similar to endothelial cells, SMCs play a crucial role in preserving the homeostasis of the ECM. In Figure 8, insulin was used as a positive control having the ability to induce hyperpolarization of SMC membranes (Zierler and Rogus, 1980; Zierler, 1966). Glucose-palmitate treatment decreased the phosphorylation levels of the insulin receptor and related pathway actors, Akt and transcription factor FOXO1 (Figure 8A). Palmitate-glucose condition also decreases glucose up-take and increases mRNA PEPCK expression, known to be under the control of FOXO1 activity (Supplementary Figure 3). Together, these data suggested that palmitate-glucose condition induced an insulin-resistance in MOVAS cells. The glucose-palmitate condition significantly increased α-SMA, MLCK, Calponin and Myh11 expressions (Figure 8B) whereas, when associated with insulin, minoxidil, or nebivolol, we observed a decrease in its expression, returning close to that of insulin alone (in the absence of insulino resistance). This suggests that *in vitro* insulino resistance could favor the contraction of MOVAS cell line, isolated from the smooth muscle of an adult mouse, by changing the levels of several genes involved in contraction. Interestingly, those expressions of “contractil genes” are associated to an increase of pro-inflammatory cytokines (TNFα, IL6 or IL1b) in insulin-resistance condition, while the presence of minoxidil or nebivolol reduces significantly the expressions of IL6 and IL1b (Supplementary Figure 3). Figures 8C, 9A show that insulin stimulates mRNA expression of elastogenesis markers (elastin, fibrillin 1, fibulin 5, LTBP4, and LOXL1) and elastin production (Figures 8D,E). Conversely, the induction of insulin resistance (despite the presence of insulin) does not elevate the expressions of these same markers or even decrease their expressions, with the possible exception of LTBP4. The addition of minoxidil or nebivolol in the presence of glucose-palmitate increases mRNA (Figures 8C, 9A) and protein (Figures 8F,G) levels of all elastogenesis markers except for LOXL1. Insulin alone has no effect on elastolysis markers, MMP-9 and cathepsin S (Figure 9B). Glucose-palmitate-induced insulin resistance significantly increased the expression of both elastases, and this effect was abolished by minoxidil or nebivolol. Surprisingly, minoxidil and nebivolol also reduced the expression of MMP-9 natural inhibitor, TIMP1 (Figure 9B). When we ratioed the expression of an elastase to that of its natural inhibitor (i.e., MMP9/TIMP1 and cathepsin S/cystatin C) (Figure 9C), we observed that insulin resistance increased the cathepsin S/cystatin C ratio, and this effect was abolished by insulin, minoxidil, and nebivolol. Conversely, glucose-palmitate reduced the MMP9/TIMP1 ratio, and neither insulin, minoxidil, nor nebivolol could modify this effect (Figure 9C). These data suggest that insulin resistance promotes MOVAS cell contraction and favors elastolysis, whereas smooth muscle relaxants, such as insulin, minoxidil, and nebivolol, are pro-elastogenic factors. In correlation studies conducted in db/db mice treated with minoxidil and nebivolol, a negative correlation was observed between contraction markers (αSMA and SM22) and elastogenic factors (elastin and LOXL1) (Supplementary Table 2).

### Minoxidil and nebivolol treatments open potassium channels in cultured vascular SMCs

Minoxidil was described as a potassium channel opener that hyperpolarizes cell membranes, causing vascular muscle relaxation and a consequent increase in blood flow (Katsuumi et al., 2018; Tam et al., 2020). The vasorelaxant effect of nebivolol has been primarily attributed to endothelial-dependent mechanisms, including beta-adrenergic receptors. However, nebivolol present additional vasorelaxant properties. Thus, the involvement of the ATP-sensitive potassium channels (KATP) of the SMCs would be a second mechanism involved in the vasorelaxant response to nebivolol (Altunkaynak-Camca, 2020; Haeusler, 1990; Kaiser et al., 2006; Korkmaz et al., 2015; Slove et al., 2013; Yasui et al., 2008). Nebivolol might appear to act indirectly on the channel by decreasing cytoplasmic ATP concentrations by inhibiting mitochondrial ATP synthase and/or increase of extracellular efflux of ATP (Supplementary Figure 4 and (Kremastiotis et al., 2021; Lafarge et al., 2010; Lafarge et al., 2014; Le et al., 2014)). The opening of potassium channels by minoxidil or by nebivolol can be associated with the induction of elastogenesis and the inhibition of elastolysis. In contrast, Supplementary Figure 5 shows that the closure of the voltage-gated potassium channels by tetraethylammonium (TEA) or of KATP channel by glibenclamide (Lebrun et al., 1997) (Lee and Dong, 2017) is associated with a decrease in most markers (protein or transcripts) of elastogenesis while those of elastolysis are expressed. To determinate if contractile status of MOVAS cells is determinant factor, we induced membrane depolarization of cells by addition of KCl in culture medium. Prior to the study, we evaluated the cytotoxic effect of extracellular KCl addition on MOVAS cell survival (Supplementary Figure 6). Excess of extracellular potassium effectively induces cell contraction but does not seem to have any significant effect on elastogenesis and elastolysis (Supplementary Figure 5). On other hand, the presence of KCl (or glibenclamide) inhibits the effects of minoxidil or nebivolol on the expressions of transcripts or proteins such as elastin. Taken together, these results suggest that the configuration of the potassium channels (opened or closed) is a major element in the control of the elastogenesis-elastolysis balance. Therefore, to determine the signaling pathway linking potassium channel and transcript expressions is important. Several studies (Hu et al., 2008; Tinker et al., 2014) suggested that the decrease of KATP channel function leads to FOXO-1 repression.

### Minoxidil and nebivolol treatments inhibit FOXO transcription factor in cultured vascular SMCs

**FIGURE 10:**
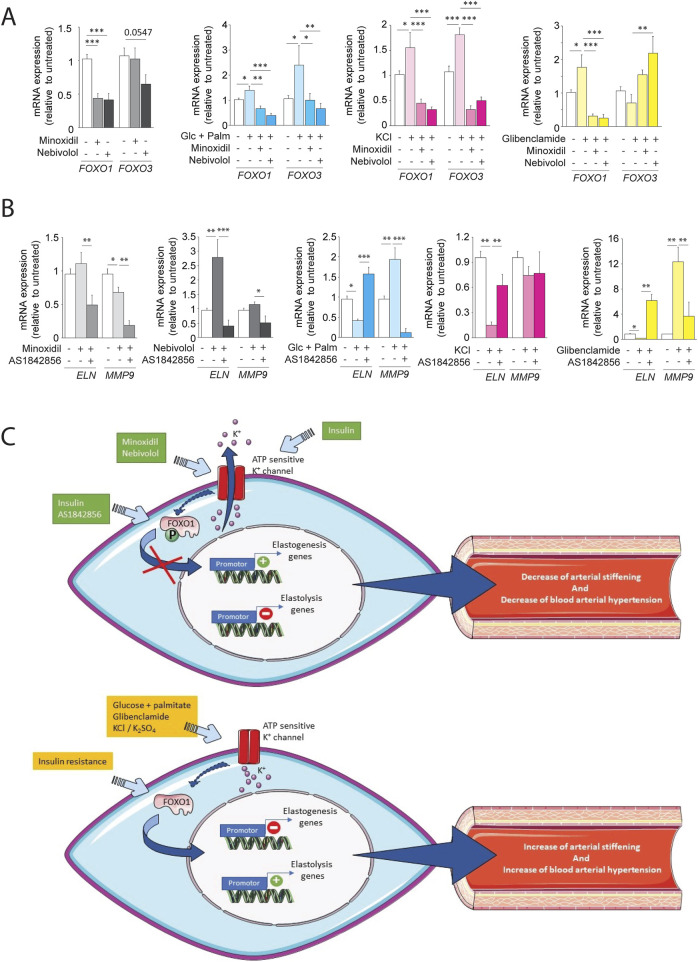
ATP-sensitive potassium channel modulation regulates FOXO transcription factors and elastin remodeling in SMCs. **(A)** mRNA expression of FOXO1 and FOXO3 in cells cultured with glucose + palmitate, KCl, or glibenclamide, ± minoxidil or nebivolol (n = 6/group); **(B)** mRNA levels of ELN and MMP-9 ± FOXO1 inhibitor AS1842856 and other conditions (n = 6/group); **(C)** Schematic model summarizing the proposed role of K^+^ channel–FOXO1 axis in the regulation of elastogenesis and elastolysis in vascular SMCsData are presented as mean ± SEM from at least three independent experiments. Statistical analysis: Kruskal–Wallis test followed by Wilcoxon–Mann–Whitney *post hoc* test with Bonferroni correction. All displayed p-values are corrected. p < 0.05 vs. untreated.

We hypothesized that the KATP channel pathway could activate the transcription factor FOXO1, involved in premature aging. Interestingly, data from the literature (Castillero et al., 2013; Nakae et al., 2001) suggest that insulin signaling pathways modulate FOXO1 activity, in accordance with our findings regarding the insulin signaling pathway (Figure 8A). In addition, we have shown that variations in elastin expression follow variations in expression and phosphorylation of FOXO1 (Figures 8F,G) depending on whether the SMCs are insulin-resistant or not or treated or not with minoxidil or nebivolol. The increased phosphorylation of FOXO associated with elastin expression was also observed in the aortas of db/db mice treated with minoxidil or nebivolol (Supplementary Figures 2A,B). Thus, we initially undertook a transcriptomic study of FOXO1 and FOXO3 on MOVAS cells incubated in different media, stimulating the opening or closing of ATP-sensitive potassium channels (see Figure 10A). In cells incubated in a classical medium (DMEM), the presence of the KATP channel openers minoxidil and nebivolol drastically reduced the expressions of FOXO1 and, for nebivolol only, FOXO3. Conversely, three KATP channels closing conditions (culture medium with glibenclamide, KCl, or glucose-palmitate) significantly increased the expression of FOXO1, whereas only KCl and glucose-palmitate elevated FOXO3 mRNA levels. KCl- or glucose-palmitate-supplemented culture medium, minoxidil, or nebivolol caused a significant decrease in FOXO1 and FOXO3 expressions compared to glucose-palmitate or KCl alone, while they decreased FOXO1 and increased FOXO3 expressions in the presence of glibenclamide compared to glucose-palmitate or KCl alone (Figure 10A). Interestingly, FOXO1 has been described as a potential regulator of elastin (Shi et al., 2012) and MMP-9 expressions (Yang et al., 2020; Zhang et al., 2017). Therefore, to confirm the impact of FOXO1 on ELN and MMP-9 expression, we used the FOXO1 transcription inhibitor AS1842856 (Figure 10B). Inhibition of FOXO1 activity by AS1842856 in insulin resistance conditions (palmitate + glucose) increased elastin and decreased MMP-9 expression compared to palmitate and glucose alone. We observed similar effects of AS1842856 on elastin and MMP-9 expressions in a glibenclamide (or KCl)-supplemented culture medium compared to glibenclamide or KCl alone. Surprisingly, in conditions where KATP channels were opened by minoxidil or in the presence of nebivolol, the FOXO1 inhibitor AS1842856 reduced the expression of MMP-9 and elastin (Figure 10B).

## Discussion

Our study demonstrates that restoring potassium channel activity via minoxidil or nebivolol significantly improves aortic wall structure and function in diabetic mice (Figure 10C). These treatments reversed key features of diabetic vasculopathy, including elastin degradation, collagen accumulation, proteolytic imbalance, and vascular stiffness (Coquand-Gandit et al., 2017; Fhayli et al., 2019; Knutsen et al., 2018; Slove et al., 2013). In insulin-resistant SMCs, both molecules enhanced elastin synthesis and FOXO1 activation, suggesting a common pathway involving potassium-dependent regulation of transcriptional activity.

These findings are consistent with the concept that diabetes and obesity induce accelerated vascular aging, which primarily manifests as extracellular matrix (ECM) degradation and arterial stiffening (Fontaine et al., 2003; Newman, 2015; Preston et al., 2018; Vanalderwiert et al., 2024b). This premature aging contributes to altered mechanical behavior of vessels (e.g., reduced compliance, increased pulse wave velocity) and impairs smooth muscle cell (SMC) contractility, contributing to long-term vascular dysfunction. Our data underscore the importance of preserving ECM homeostasis to mitigate both structural and functional decline. Indeed, from adolescence to death, an individual lives with a defined stock of EFs, which is progressively degraded (Wagenseil and Mecham, 2009). Indeed, elastogenesis begins *in utero* and stops at the end of childhood in mice and humans. During adulthood, repair or neosynthesis of EFs is no longer possible. Nevertheless, in getting older, fibers are subjected to mechanical stresses of stretching-relaxation and possible inflammatory attacks following the release of elastases. This fatefully leads to a slow and progressive degradation of EFs architecture (Duca et al., 2016). During chronic inflammatory pathologies, such as obesity or diabetes, a chronic elastolytic activity (e.g., cathepsin S and NE) prevails, which may explain the premature fragmentation of EFs (Duca et al., 2016). Finally, this elastin degradation and the ensuing EDP release are considered as the aging markers and contribute to cardiovascular diseases such as arterial hypertension, atherosclerosis, and aneurysms (Boutouyrie et al., 2002; Reference Values for Arterial Stiffness C, 2010; Sarafidis et al., 2017) but also contributes to metabolic syndrome development (Blaise et al., 2013; Hocine et al., 2020; Romier et al., 2018). Preserved EF storage or induced a neosynthesis of EF in aorta MEC is a major question to limit those cardio-metabolism diseases.

While multiple classes of antihypertensive agents (ACE inhibitors, ARBs, calcium channel blockers) provide hemodynamic benefit, their impact on vascular structure—especially elastic fiber homeostasis—is limited or indirect (Le et al., 2014; Pickkers et al., 1998; Shahin and Johnson, 2016; Wojakowski et al., 2001; Zhou et al., 2019). Previous studies (Lebrun et al., 1997; Nakano et al., 2002) have shown that only certain drug classes, such as β3-agonists or K_ATP channel openers, exert favorable effects on ECM remodeling. Minoxidil, although potent, is limited by systemic side effects (Olawi et al., 2019; Toblli et al., 2010; Wang et al., 2009; Wojciechowski and Papademetriou, 2008). Our mechanistic results suggest that both drugs promote elastogenesis via activation of FOXO1, a transcription factor linked to metabolic and redox homeostasis (Lee and Dong, 2017). Potassium channel modulators (minoxidil, nebivolol) maintained FOXO1 activation under insulin-resistant and high-glucose/high-fat conditions. Pharmacological inhibition of FOXO1 abrogated this effect. Although we did not perform direct electrophysiological measurements (e.g., patch-clamp), the use of modulators such as KCl and glibenclamide supports the involvement of K_ATP and voltage-gated K^+^ channels. However, as acknowledged, prolonged KCl exposure may also activate voltage-gated calcium channels, a possible confounder to be addressed in future work.

Importantly, our findings also suggest that minoxidil and nebivolol may attenuate vascular senescence. We observed decreased expression of SASP markers (IL-6, IL-1β), along with reduced protease expression (NE, cathepsin S) and increased expression of their natural inhibitors (TIMP1, SERPIN). These molecular changes, associated with improved ECM integrity, strongly suggest an anti-senescent effect, consistent with our previous report on the presence of vascular senescence in db/db mice (Vanalderwiert et al., 2024b). Moreover, several studies (Martins et al., 2016; Morris et al., 2015) suggest that members of the FOXO family play a major role in longevity by increasing the expression of genes involved in defense anti-stress activity, metabolism, and cell cycle arrest.

This study has several limitations. First, electrophysiological recordings were not performed, preventing direct confirmation of ion channel modulation. Second, only male mice were used, limiting conclusions regarding potential sex-specific responses. Third, the study design was cross-sectional and did not assess long-term or reversible effects. Nevertheless, the consistency between *in vivo* and *in vitro* findings supports the robustness of the observed mechanisms.

From a translational standpoint, targeting FOXO1 via upstream modulators represents an attractive strategy to combat diabetic vasculopathy. Transcription factors like FOXO1 are classically difficult to target directly, but modulating potassium channels offers a viable alternative. Indeed, data in the literature (Raeis et al., 2010) suggest that ATP-sensitive potassium channel signaling pathway involves the transcription factors FOXO1, FOXO3. Moreover, β-adrenergic agonists can increase the expression and/or activity of members of the FKHR family (Lynch and Ryall, 2008) while this same factor controls the expression of elastin (Shi et al., 2012). Therefore, nebivolol’s safety profile and endothelial benefits may make it a particularly promising candidate for clinical application. Further studies in diabetic patients or vascular tissue from human donors will be essential to confirm these mechanistic links and their therapeutic potential. The control of the expression of the transcription factor FOXO would thus be an interesting alternative therapeutic target to prevent physiological or premature aging, as observed in diabetes and/or obesity.

Beyond the primary mechanisms and therapeutic effects described above, several additional considerations warrant discussion. In addition to the mechanical and structural improvements in the aortic wall, both treatments significantly modulated inflammation and ECM degradation pathways. We observed a reduction in SASP-associated cytokines (e.g., IL-6, IL-1β), as well as decreased activities of elastolytic enzymes (e.g., NE, cathepsin S), paralleled by an increase in their endogenous inhibitors (e.g., TIMP1, SERPIN) (Coppe et al., 2010; Katsuumi et al., 2018; Narasimhan et al., 2021; Nerstedt and Smith, 2023; Safwan et al., 2022). These changes, coupled with restored ECM architecture, suggest that both minoxidil and nebivolol may exert anti-senescent vascular effects. Although we did not directly assess senescence via SA-β-gal or p16/p21 expression, the downregulation of SASP components supports a reduction in senescence burden (Coppe et al., 2010; Katsuumi et al., 2018; Narasimhan et al., 2021; Nerstedt and Smith, 2023; Safwan et al., 2022). This complements previous reports that diabetic vasculopathy is driven not only by ECM degradation, but also by chronic low-grade inflammation and premature cellular aging (Vanalderwiert et al., 2024).

However, our study was limited to male db/db mice, and sex-specific differences in vascular aging, inflammation, and metabolic response have been widely reported. For instance, estrogen signaling influences SMC phenotype and ECM synthesis, and females may exhibit delayed or distinct patterns of vascular remodeling in diabetes (Chen et al., 2016; Sivasinprasasn et al., 2017; Xin et al., 2002). The lack of female data limits extrapolation to the broader diabetic population. Future studies in both sexes are essential to evaluate whether the observed FOXO1–K^+^ axis and treatment effects are conserved across biological contexts.

Finally, while our results identify FOXO1 as a promising regulatory node in vascular aging, its direct targeting remains challenging due to its transcription factor nature, which complicates pharmacological inhibition or activation. Off-target effects, nuclear localization dynamics, and pathway crosstalk also pose hurdles for clinical translation. However, the use of upstream modulators—such as potassium channel openers or β3-agonists—provides an indirect and potentially safer route. Nebivolol, already in clinical use, represents a viable candidate with established safety data, though further studies are needed to evaluate its long-term impact on vascular aging endpoints in diabetic patients.

## Data Availability

The original contributions presented in the study are included in the article/Supplementary Material, further inquiries can be directed to the corresponding author.

## Abbreviations

αSMA, α-smooth muscle actin; AFM, Atomic Force Microscopy; Cath. S, cathepsin S; Dex, dexamethasone; DMEM, Dulbecco’s Modified Eagle Medium; ECM, ExtraCellular Matrix; EF, Elastic Fiber; ELN, Elastin; FBLN5, fibulin-5; FBN1, fibrillin-1; FOXO, Forkhead box O; LOXL1, lysyl oxidase L1; LTBP4, latent transforming growth factor beta binding protein 4; MAP, Mean arterial pressure; MMP, Matrix Metalloproteinase; NE, neutrophil elastase; SERPIN, serine proteinase inhibitor; SM-22α, Smooth muscle protein 22-alpha; SMC, smooth muscle cell; VSMC, vascular smooth muscle cell; TEA, tetraethylammonium ion; TIMP1, Tissue inhibitor of metalloproteinases.
