# Supplementary material for: Minoxidil and nebivolol restore aortic elastic fiber homeostasis in diabetic mice via potassium channel activation
Source: Front Physiol. 2025 Sep 18;16:1648727. doi: 10.3389/fphys.2025.1648727 (PMC12488662; doi:10.3389/fphys.2025.1648727)
Supplement: Supplementary file 1 [file DataSheet1.pdf]

Minoxidil and Nebivolol Restore Aortic Elastic Fiber Homeostasis in Diabetic Mice via Potassium Channel Activation

Auberi Henry[1#], Laetitia Vanalderwiert[1#], Amandine Wahart[1], Daniel A Carvajal Berrio[2], Eva M Brauchle[2] [2,3], Katja Schenke-Layland[2,3,4], Juergen Brinckmann[5], Heiko Steenbock[5], Laurent Debelle[1], Isabelle Six[6], Gilles Faury[7], Stéphane Jaisson[1,8], Philippe Gillery[1,8], Vincent Durlarch[1,9], Hervé Sartelet[1], Pascal Maurice[1], Amar Bennasroune[1], Laurent Martiny[1], Laurent Duca[1], Béatrice Romier[1], Sébastien Blaise[1*]

SUPPLEMENTAL MATERIAL

High-frequency ultrasound imaging – High-resolution ultrasound imaging was performed with animals anesthetized (isoflurane 4%). The animals were depilated with hair removal cream and placed on a heated table (37°C). For ultrasound measurements, a Vevo3000 ultrasound imaging system (VisualSonics, Toronto, Canada) with a 30 MHz linear signal transducer was used for measurements of anatomical and functional parameters of the left ventricle of the heart needed to determine factors such as ejection fraction, cardiac output, fractional area change, fractional shortening, stroke volume, end-systolic volume (LVESV), and end-diastolic volume (LVEDV).

Histology – Dissection of the aorta: The descending thoracic aorta was removed without "cleaning" of the perivascular fat to preserve the integrity of the structures (adventitia and media). This approach allows us to avoid tearing off the tunica adventitia and creating ruptures of the EFs within the tunica intima-media, consecutive to the withdrawal of perivascular adipose tissue. The aorta samples were embedded in paraffin. Three aorta cross-sections from three different animals in each group were stained with hematoxylin–eosin (H&E), Picrosirius red for collagen staining, and Hart's staining in accordance with Davis and Li [1]. Picrosirius red staining was observed microscopically under nonpolarized or polarized light (Leica, Paris, France). Using tissue stained with H&E and ImageJ software (National Institutes of Health), the intimal-medial thickness was measured from the internal elastic lamina to the external elastic lamina at three independent regions of interest per quadrant from each sample, according to Balint *et al*. [2] and Romier *et al*. [3]. The intima-media mean thickness was calculated for each animal and compared between the groups. Collagen deposition on the adventice layer was measured with ImageJ software through quantification of Picrosirius red staining. According to Balint *et al*. [2], the color deconvolution plug-in was used to separate the color channels corresponding to collagen for each stain, and the area of collagen was calculated relative to the vessel area. Regarding EFs, three independent researchers who worked blinded to the sample groups and counted lamellar ruptures and lamella number (n=10 ROIs per quadrant of each sample).

Immunofluorescent staining – The expression of cellular and ECM proteins was assessed by immunolabeling the aortic tissue sections (4 µm) with primary antibodies targeting alpha-Smooth Muscle Actin (α-SMA) (Santa Cruz Biotechnology, Dallas, TX, USA). Elastin autofluorescence in tissue sections was recorded with excitation at 488 nm, according to the method of Romier *et al*. [3]. Image acquisitions were achieved through microscopy (Olympus, Paris, France). Analysis of positive pixel/mm^2 of images (n=4 regions of interest per sample) was performed with ImageJ software

Cell culture –MOVAS cell line (ATCC, Manassas, USA), derived from aortic smooth muscle cells of male C57BL/6J mice, was cultured in DMEM containings high glucose (4.5 g/L, Gibco Life Technologies, Paisley, United Kingdom) supplemented with fetal calf serum (FCS 10%) (PAN Biotech, Aidenbach, Germany) and geneticin (G418 0.2 mg / mL) (Gibco). The maximum passage where cells are used, is 5. The cells were incubated with 20 mM of glucose and 0.5 mM of palmitate (Sigma, St. Louis, USA) for 48h [4, 5] to induce an insulin-resistance. Once insulin-resistant, MOVAS cells were then incubated for 48 h with culture medium containing 10 µM minoxidil (a K_{ATP} channel opener [6]) or 10 µM nebivolol (a β-blocker also favoring K_{ATP} channel opening [7]) or PBS. To determine the role of potassium channels, MOVAS cells were incubated for 48 h with either glibenclamide (10 µM, a K_{ATP} channel blocker [8]), tetraethylammonium (TEA, 50 mM, a non-specific blocker of voltage-gated K^+ channel [9]) or KCl (50 mM, which depolarizes the cell membrane). In the case of glibenclamide, at 10 µM, the molecule caused a reversible 24 mV depolarization in the myocyte of aorta [10] while inactivation of the K^+ channel by TEA increased the membrane potential to +50 mV [9, 11]. In the case of KCl, the Goldman equation indicated a tenfold change in extracellular K^+ from 5 to 50 mM, which causes 48 mV membrane depolarization under physiological conditions [12, 13]. According to Zhou *et al*. [14], MOVAS cells were incubated with AS1842856 (1 µM, Sigma) to inhibit FOXO1 activity.

Western Blot - was performed as previously described by Blaise et al. [15] . Proteins were obtained after homogenization of frozen tissue in the following Buffer (50 mM Tris-Cl pH 7.5, 100 mM NaCl, 5 mM EDTA, 5 mM EGTA, 1 mM DTT, 0,5% Triton X-100, 2 mM PMSF) supplemented with complete protease inhibitor tablet (Roche, France) to avoid protein degradation. Protein homogenates were mixed with 4x Laemmli buffer, boiled 5 min at 95°C, separated on 10 % SDS polyacrylamide gels and transferred onto nitrocellulose membranes. After blocking the membranes overnight at 4 °C in 5% milk in TBST, primary antibodies were applied at the specific dilution in 5% milk in TBST buffer and incubated for 1 h at RT or overnight at 4°C depending on the specificity of the antibodies and manufacturer's instructions. After adding the primary antibody, the membranes were washed 5 times with TBST, and horseradish peroxidase-conjugated secondary antibodies (Cell Signaling, UK) were applied for 1 h. After 5 times washing with TBST, membranes were developed using superSignal west Pico kit (Thermo scientific, PEIRCE) and ODYSSEY Fc (Lycor) hardware. The antibodies used for western blot were, the rabbit polyclonal anti- FOXO1, p-FOXO1 (T24), and Akt, p-Akt (S473 and T308) from cell signaling, rabbit polyclonal anti-Elastin (BA4, Sigma). The antibodies against insulin receptor (IR), its phosphorylated (p-IR), and anti-actin were purchased from Santa Cruz Biotechnology (Dallas, USA). The quantification was determined using the ImageJ software measuring by the gray level of the western blot.

Gene expression was analyzed by qPCR, as previously described [3]. Total RNA was extracted using Trizol reagent (Eurobio Scientific, Les Ulis, France). The RNA concentration was measured using a NanoDrop system (ThermoFisher Scientific, Illkirch, France). The 260/280 ratio was calculated, using NanoDrop software, to evaluate protein contamination. Complementary DNA (cDNA) was generated using a Verso cDNA kit (Thermo Scientific, Illkirch, France). Real-time PCR was performed using SYBR Green on a BioRad CFX96 Real-Time System (BioRad, Hercule, California, USA). In this study, 5 µl cDNA (1/10) and 0.7 µl of each forward and reverse primer (3 µM) were used for the qPCR test, with cycling conditions as follows: 95°C for 15 minutes, 40 cycles of 95°C for 10 seconds, and 60°C for 60 seconds.

RNA expression was normalized to the housekeeping genes 36B4 and RPS26, and relative gene expression was calculated using the $2^{-\Delta\Delta CT}$ method. All RNA expressions are presented relative to those of the control model of non-diabetic C57Bl6 mice. A dashed red line will represent these non-diabetic mice. Supplementary Table 1 presents the forward and reverse sequences.

SUPPLEMENTAL FIGURES 1 to 10 AND SUPPLEMENTAL TABLE1

Supplemental table 1: Sequences used for qPCR.

Gene name	Forward sequence (5’ → 3’)	Reverse sequence (5’ → 3’)
RPS26	TAGAAGCCGCTGCTGTCAGG	GGCACAGCTCACGCAATAATG
36B4	AAAGCCTGGAAGAAGGAGGTC	AGATTCGGGATATGCTGTTGG
MMP-9	CACGGAGACGGGTATCCCTT	GGGCACCATTTGAGTTTCCAT
Myosin light-chain kinase (M*LCK)*	TGGGGGACGTGAAACTGTTTG	GGGGCAGAATGAAAGCTGG
Neutrophil elastase (NE)	TGGAGGTCATTTCTGTGGTG	CTGCACTGACCGGAAATTTAG
Cathepsin-S	GCGTCACTGAGGTGAAATACC	CCCCCACAGCACTGAAAG
Cystatin C	ATGACCAGCCCCATCTGA	CCAGGGCACGCTGTAGAT
Tissue inhibitors of metalloproteinase (Timp1)	TCCCCAGAAATCAACGAGACC	GTACCGGATATCTGCGGCATT
Serine peptidase inhibitor (SERPIN)	TAGGGAGCAAGGGTGACACTC	ACTGTCTGGTCTGTTGAGGGT
Elastin (ELN)	GCTGCTGCTAAGGCTGCTAA	AGCACCTGGGAGCCTAACTC
Fibrillin 1 (FBN1)	GGACGGAAAGAACTGTGAAGAT	ACACATTCCGTTTAGGCACA
Fibulin 5 (FBLN5)	ATCTGCTGATTGGTGAAAACC	ATGGTGAATGGCTGGTCTCT
Lysyl oxidase like 1 (LOXL1)	GAATACTGAGCCAGACTGGC	GGGTCTCATTGAAATTAGTATCC
Lysyl oxidase like 2 (LOXL2)	ATGACCTGCTGAGCCTCAAC	CAGTGTCCTCCAGGCAGAAG
Lysyl oxidase like 3 (LOXL3)	ATGGGTGCCATCCACTTGAG	TGTTCTTGGACGGGCATCTC
Lysyl oxidase like 4 (LOXL4)	GGTTGTGAACCCCACAAACG	CTGCATTGGCTCGGTAGGAA
Lysyl oxidase (LOX)	CTATTCGATCCCACGCTGCT	CCTCACAATGGGGATGTGCT
Latent TGF beta binding protein (Ltbp-4)	CGTCAACGAGTGTGATGAGG	GAGCAAATCCTGGACGACAG
Smooth mucle actin (α—SMA)	ACTGGTATTGTGCTGGACTCTG	TAGTCACGAAGGAATAGCCACG
Smooth muscle (SM22α)	CCCAGACACCGAAGCTACTC	GACTGCACTTCTCGGCTCAT
h-caldesmon	TACACCAATGCAATCGAGGGAA	TACATCTCCTGGCCTCAAGTCA

Supplementary table 2: Relationship between vasocontraction factors and elastogenesis factors in db/db mice treated or not with minoxidil or nebivolol.

mRNA expression	mRNA expression	correlation	Z score	P-value	Range (inf 95% - sup 95%)
h-Caldesmone	LTPB4	0.726	4.598	**<0.0001**	0.484 – 0.865
	LOXL1	0.603	3.489	**0.0005**	0.297 – 0.797
	FBLN5	0.551	3.099	**0.0019**	0.224 – 0.766
	FBN1	0.401	2.123	**0.0338**	0.033 – 0.673
	ELN	0.793	5.399	**<0.0001**	0.597 – 0.900
αSMA	LTPB4	- 0.492	- 2.694	**0.0071**	- 0.731 - -0.146
	LOXL1	- 0.588	- 3.375	**0.0007**	- 0.788 - -0.276
	FBLN5	- 0.138	- 0.695	0.4870	-0.486 – 0.248
	FBN1	- 0.028	0.138	0.8900	-0.349 – 0.397
	ELN	- 0.718	- 4.520	**<0.0001**	-0.861 - -0.471
SM22α	LTPB4	- 0.526	-2.921	**0.0035**	- 0.751 - -0.190
	LOXL1	- 0.538	-3.004	**0.0027**	-0.759 - -0.206
	FBLN5	- 0.161	-0.811	0.4175	-0.504 – 0.226
	FBN1	- 0.151	0.754	0.4510	-0.237 – 0.495
	ELN	- 0.597	-3.444	**0.0006**	-0.794 - -0.288

αSMA, alpha- smooth muscle actin - SM22a, smooth muscle 22alpha- LTBP4, Latent Transforming Growth Factor Beta Binding Protein 4 - LOXL1, Lysyl Oxidase Like 1 – FBLN5, Fibulin 5 – FBN1, Fibriline 1 – ELN, Elastin

Supplemental Table S3. Baseline values from C57Bl/6 control mice

Parameter	Unit	Mean	SEM	n
**Systolic Blood Pressure**	mmHg	124.2	0.8	10
**Diastolic Blood Pressure**	mmHg	48.3	1.1	10
**Pulse Wave Velocity**	m/s	2.37	0.12	10
**Young modulus of aorta**	MPa	0.09	0.02	10
**Intima -Media thickness**	µm	57.4	3.6	10
**Advencia thickness**	µm	32.1	3.5	10
**Elastin content**	µg/mg tissue	23.5	3.6	10
**Total crosslink**	pmol/µg of elastin	24.0	0.4	
**Collagen quantity**	µg/mg tissue	21.7	0.6	5
**Total crosslink**	mol/mol of collagen	0.78	0.01	5
**Young modulus of elastic fibers**	MPa	0.40	0.03	10

Values represent mean ± SEM from C57Bl/6 mice used as visual references (dashed red lines) in the main figures. All groups (C57Bl/6, db/db untreated, db/db + minoxidil, db/db + nebivolol) were studied concurrently under identical experimental conditions. These C57Bl/6 values served two distinct purposes in our analysis: - For physiological, histological, and crosslinking measurements (e.g., blood pressure, ultrasound, collagen and elastin crosslinks, picrosirius staining, elastin autofluorescence), the values were presented and compared using **raw (absolute) data**. - **For molecular and biochemical endpoints** (e.g., mRNA expression, elastase activity, plasma desmosine and EDP, inflammatory factors), the values from db/db mice (treated or not) were **normalized to the mean C57Bl/6 value**, which was arbitrarily set to 100% (or 1), in order to highlight percentage changes from physiological baseline.

A broader phenotypic characterization of both C57Bl/6 and untreated db/db mice—including raw values for all endpoints—was published in Vanalderwiert et al. (*Am J Physiol Cell Physiol*, 2024; doi: 10.1152/ajpcell.00615.2023).

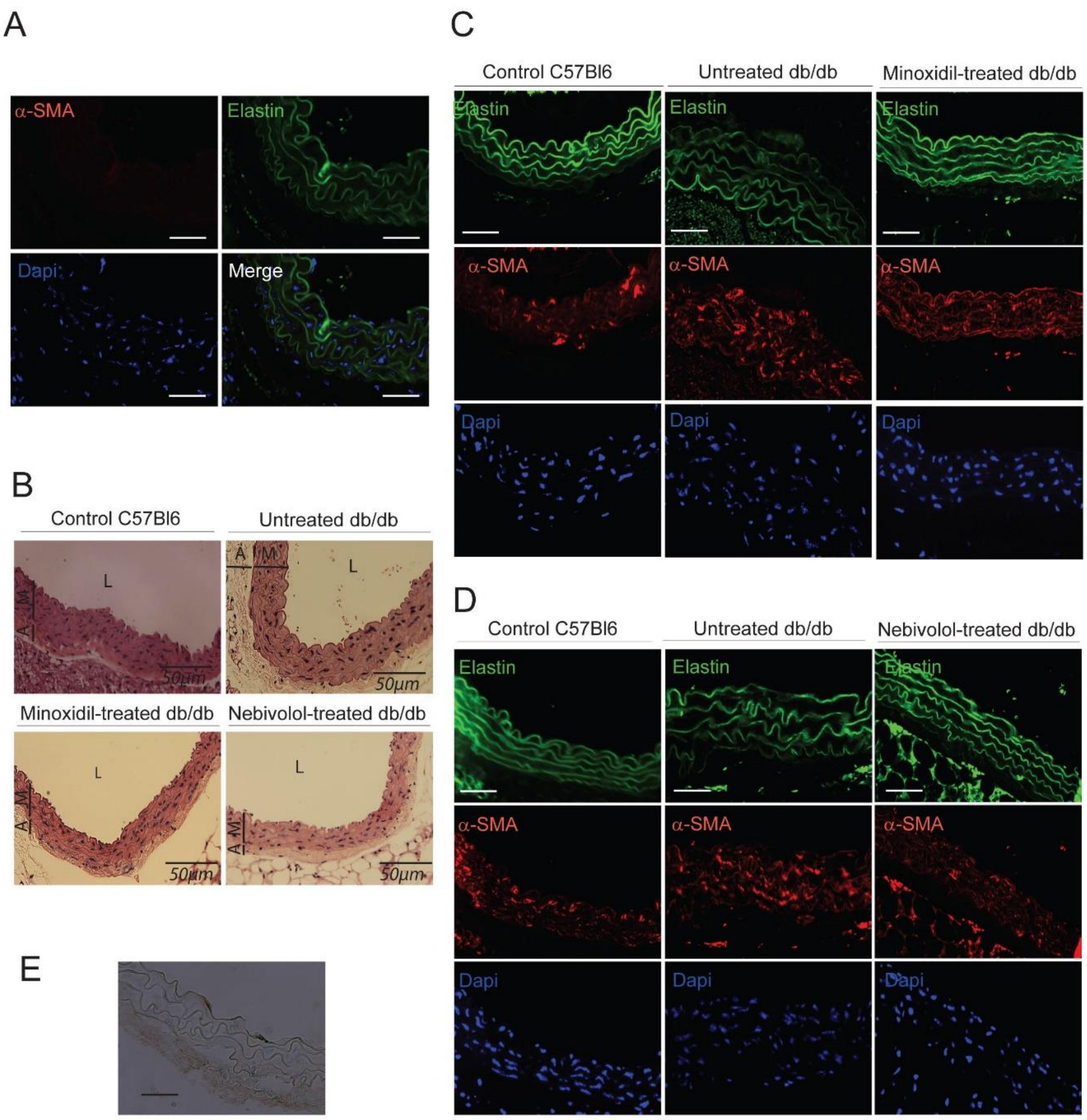

Supplemental data 1

Supplemental Figure S1.

(A) Negative control of immunostaining for α-smooth muscle actin (α-SMA, red). Elastin autofluorescence appears in green and DAPI staining in blue.

(B) Representative hematoxylin–eosin (H&E) staining showing intima–media thickness (M) and adventitia thickness (A). Scale bar: 50 µm.

(C, D) Immunostaining for α-SMA (red), elastin autofluorescence (green), and DAPI staining (blue) in aortic media from untreated db/db mice or mice treated with minoxidil (C) or nebivolol (D).

(E) Negative control for Hart's staining.

.

Supplemental data 2

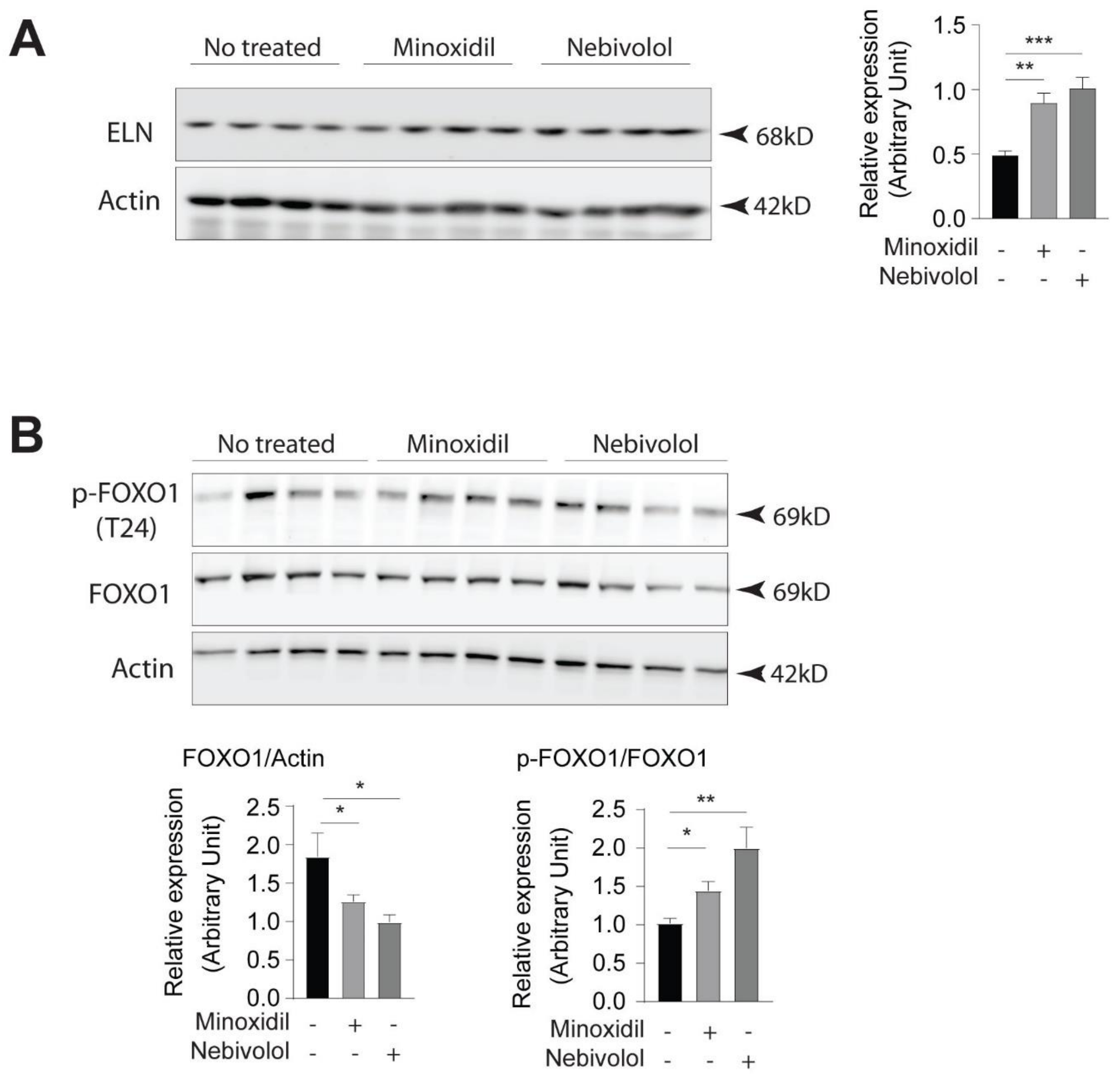

Supplemental Figure S2 - Impact of minoxidil and nebivolol treatments on elastin (ELN) expression and FOXO1 activity in vivo in treated or untreated db/db mice (n = 4 per group).

(A) Representative Western blots for elastin. Quantification of elastin expression was performed using ImageJ software to analyze gray-level intensity.

(B) Representative Western blots for FOXO1 and its phosphorylated form. Quantification of

expressions was performed using ImageJ software to analyze gray-level intensity.
Data are expressed as mean ± SEM. Statistical significance was determined using the Mann–Whitney test with Bonferroni correction for multiple comparisons. Adjusted p-values are indicated as *** $p < 0.0001$, ** $p < 0.001$, * $p < 0.05$.

Supplemental data 3

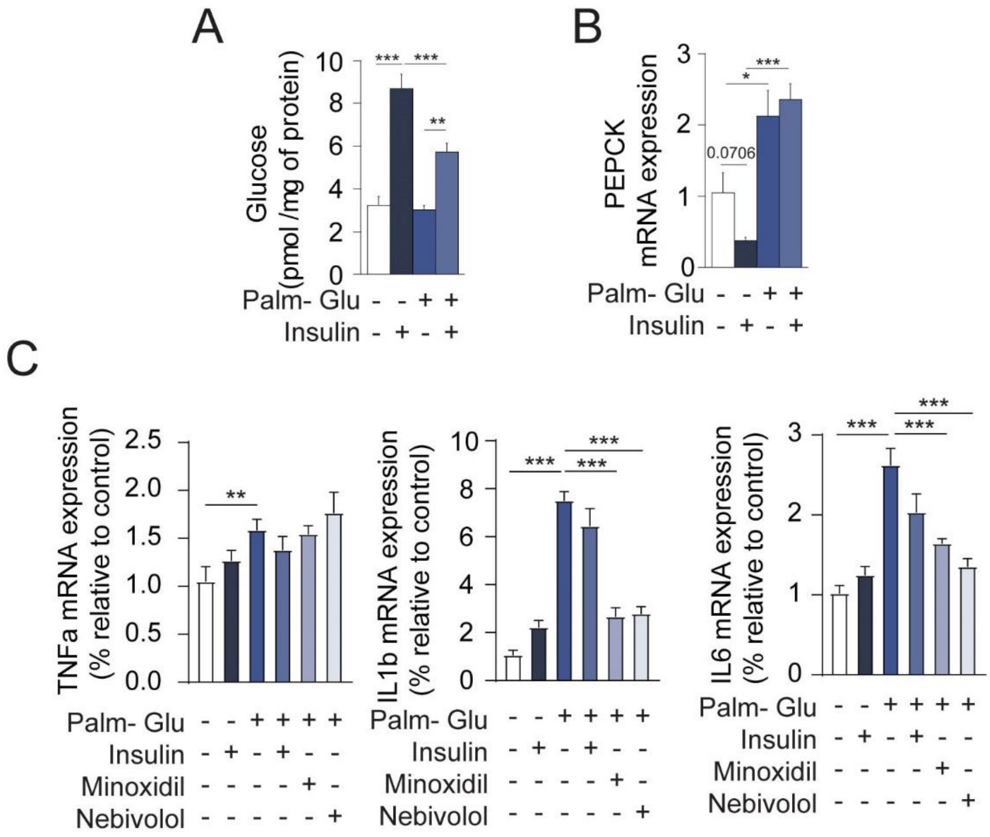

Supplemental Figure S3 - MOVAS cells incubated with palmitate and glucose develop insulin resistance, characterized by:

(A) Reduced glucose uptake.

(B) Increased mRNA expression of phosphoenolpyruvate kinase, determined by qPCR.

(C) mRNA levels of inflammatory markers from aortic tissue (n = 10 per group).

(D) Expression levels of inflammatory markers in cell culture medium, quantified by ELISA (n = 5 per group).

Data are presented as mean ± SEM. Statistical significance was assessed using the Mann–Whitney test with Bonferroni correction for multiple comparisons. Adjusted p-values are indicated as *** $p < 0.0001$, ** $p < 0.001$, * $p < 0.05$.

Supplemental figure 4

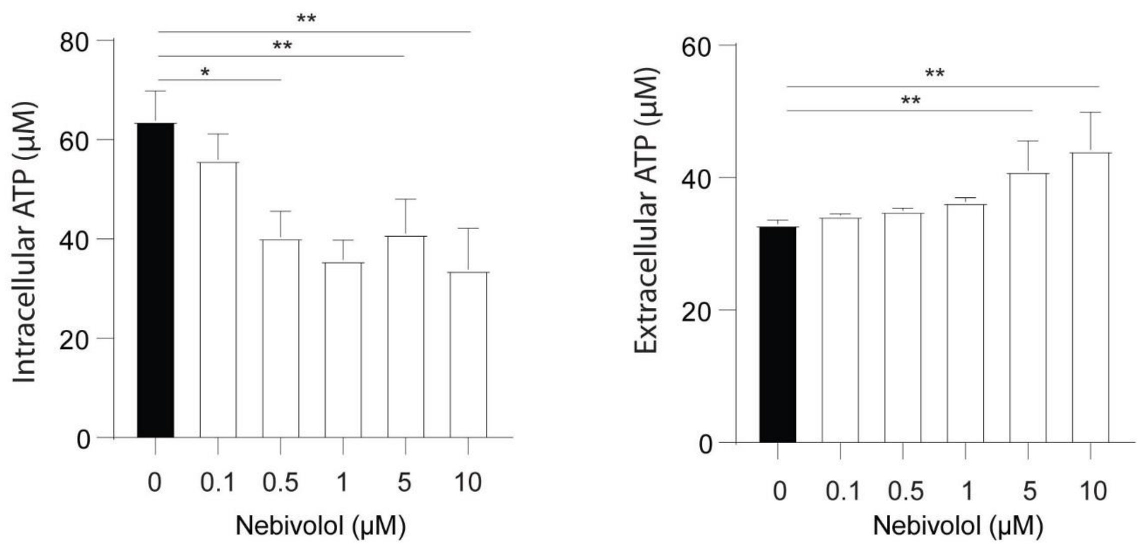

Supplemental Figure S4 - Intracellular and extracellular ATP concentrations in MOVAS cells stimulated with nebivolol (n = 4 per group).

Data are presented as mean ± SEM. Statistical significance was assessed using the Mann–Whitney test with Bonferroni correction for multiple comparisons. Adjusted p-values are indicated as ** $p < 0.001$, * $p < 0.05$.

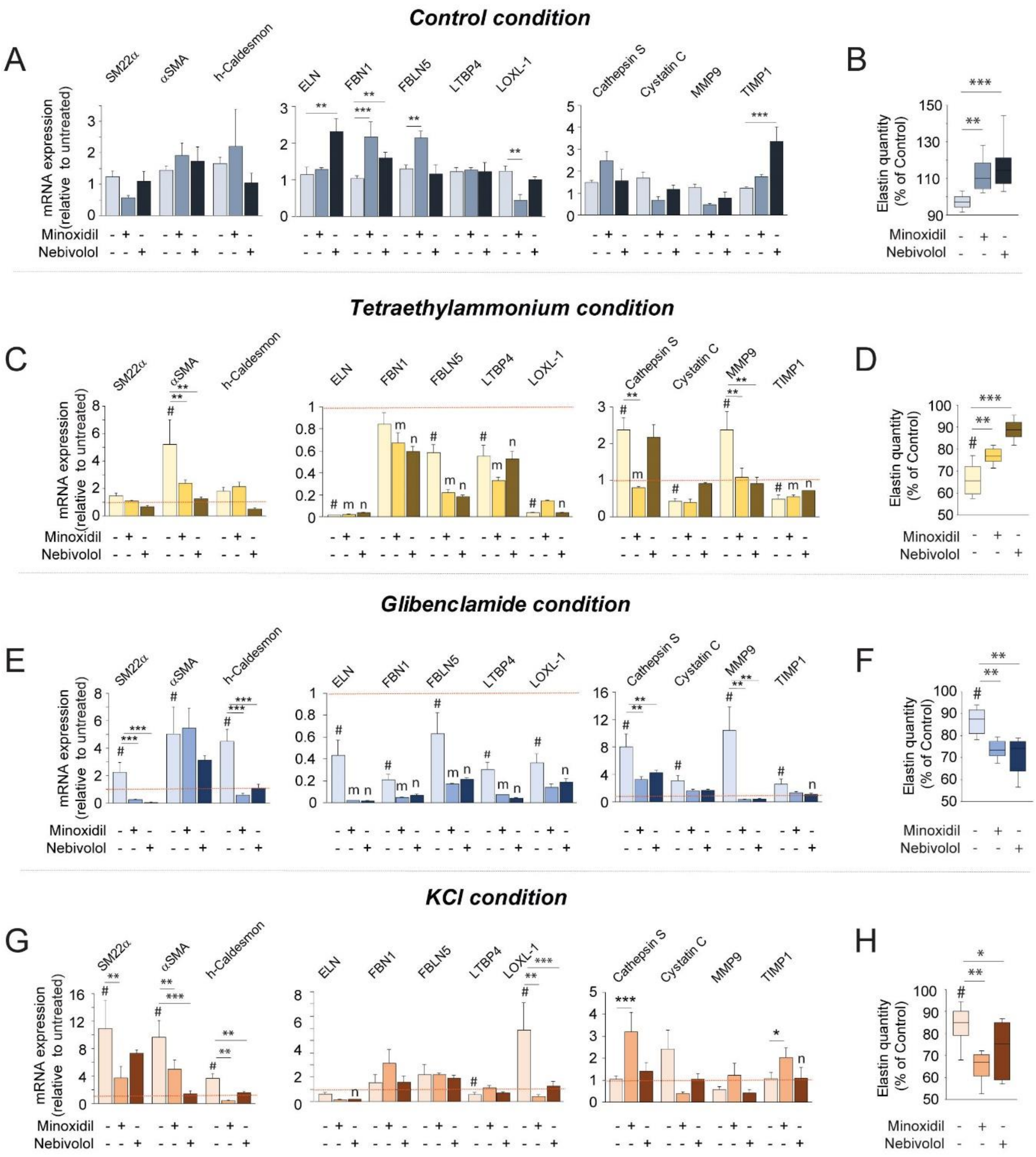

Supplemental Figure 5

Supplemental Figure S5 - Modulation of the elastogenesis–elastolysis balance in cultured vascular smooth muscle cells (MOVAS) by changes in extracellular K^+ concentration and stimulation with K^+ channel openers.

(A, B) Effect of minoxidil or nebivolol alone on mRNA expression of contractile markers (α-SMA, SM-22α, h-caldesmon), elastogenesis markers (elastin [ELN], fibrillin-1 [FBN1], fibulin-5 [FBLN5], latent transforming growth factor beta binding protein 4 [LTBP4], lysyl oxidase-like 1 [LOXL1]), and elastolysis markers (MMP-9, cathepsin S) along with their endogenous inhibitors (TIMP1, cystatin C) (A), and on extracellular elastin protein levels measured by ELISA (B).

(C–F) Effect of non-selective K^+ channel inhibition by tetraethylammonium (C, D) or selective K_ATP channel inhibition by glibenclamide (E, F), alone or combined with minoxidil or nebivolol, on mRNA

expression of the same contractile, elastogenesis, and elastolysis markers (C, E) and on extracellular elastin protein levels (D, F).

(G, H) Effect of extracellular K^+ accumulation (50 mM KCl for 48 h), alone or combined with minoxidil or nebivolol, on mRNA expression (G) and extracellular elastin protein levels (H).

For mRNA data, results are normalized to the basal condition (DMEM alone, no treatment) and expressed relative to this reference (red dotted line in C, E, G, H). For protein data (B, D, F, H), results are normalized to the basal condition set at 100%.

n = 3 per group. Data are presented as mean ± SEM. Statistical significance was determined using the Mann–Whitney test with Bonferroni correction for multiple comparisons. Adjusted p-values are indicated as *** $p < 0.0001$, ** $p < 0.001$, * $p < 0.05$. Symbols #, m, and n indicate $p < 0.05$ versus the basal condition (mRNA values in panel A).

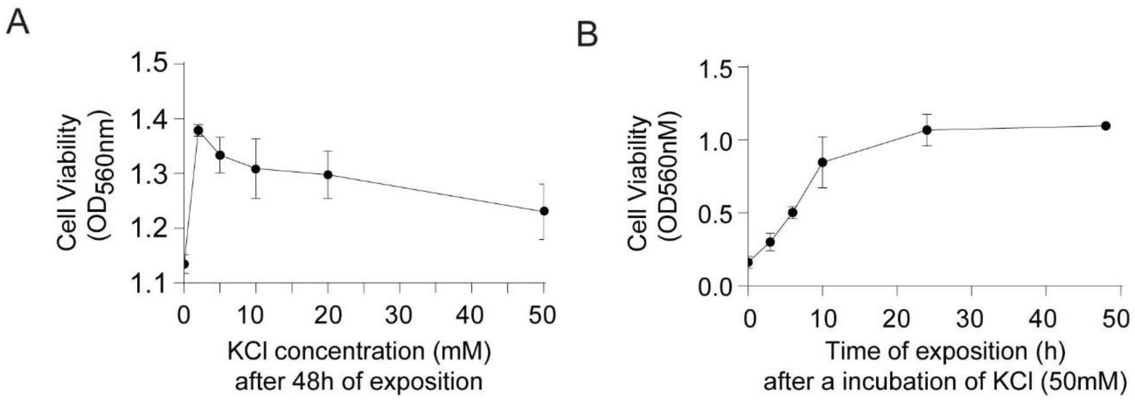

Supplemental figure 6

Supplemental Figure S6 - In vitro evaluation of the effect of KCl on the viability of vascular smooth muscle cells (MOVAS).

(A) Cell viability after 48 h incubation with increasing concentrations of KCl (0–50 mM).
(B) Cell viability after exposure to 50 mM KCl for durations ranging from 2 minutes to 48 h.

n = 3 per condition. Data are presented as mean ± SEM. Statistical significance was determined using the Mann–Whitney test with Bonferroni correction for multiple comparisons. Adjusted p-values are indicated as *** $p < 0.0001$, ** $p < 0.001$, * $p < 0.05$.

Full Membrane for Western blot

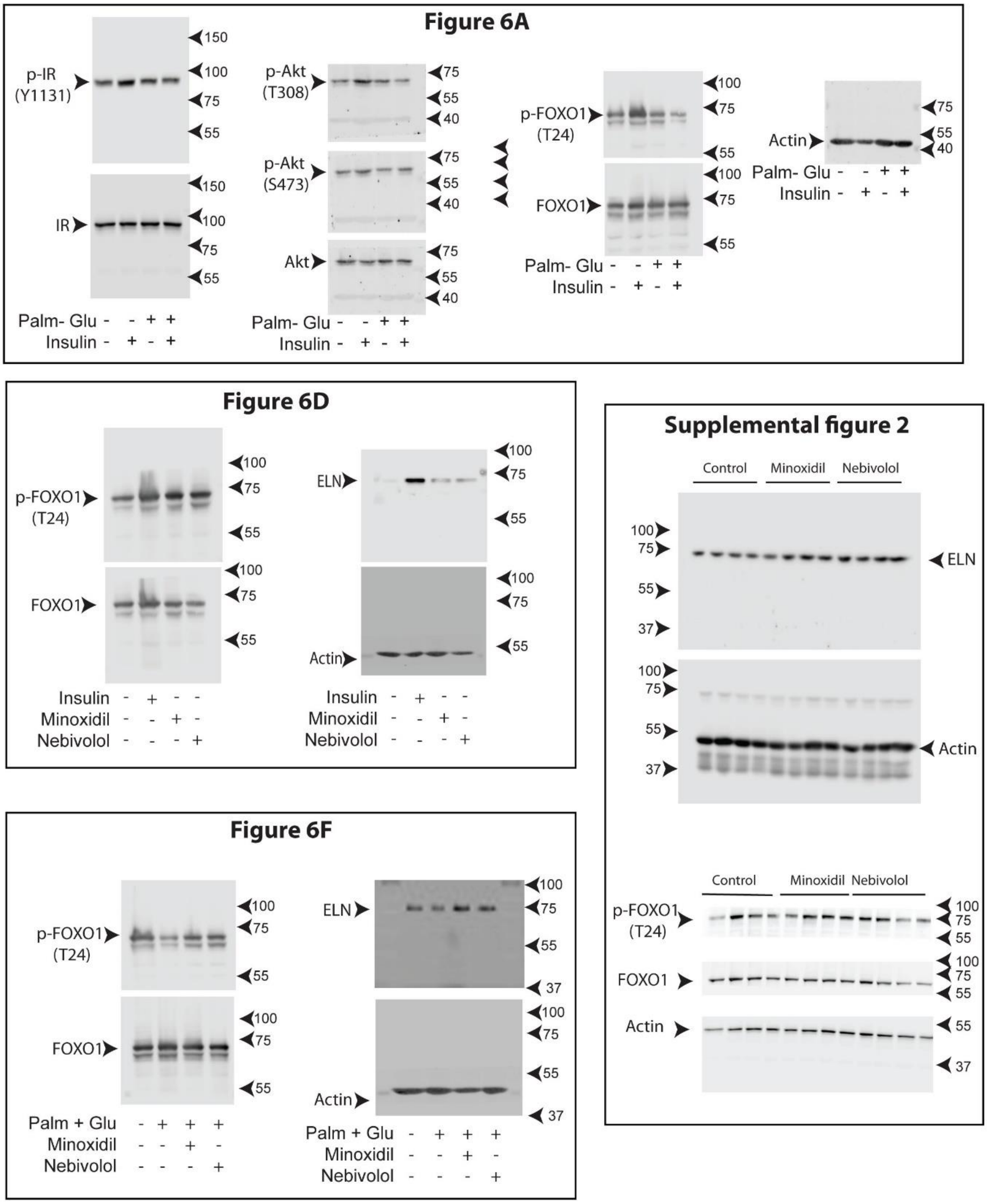

Supplemental Figure 7: Full membranes of western blot presented in figure 6 and in supplemental figure 2.

1. Davis EC, Li L: **Histological and Electron Microscope Staining for the Identification of Elastic Fiber Networks**. *Methods Mol Biol* 2017, **1627**:385-393.
2. Balint B, Hergalant S, Camadro JM, Blaise S, Vanalderwiert L, Lignieres L, Gueant-Rodriguez RM, Gueant JL: **Fetal Programming by Methyl Donor Deficiency Produces Pathological Remodeling of the Ascending Aorta**. *Arterioscler Thromb Vasc Biol* 2021, **41**(6):1928-1941.
3. Romier B, Dray C, Vanalderwiert L, Wahart A, Hocine T, Dortignac A, Garbar C, Garbar C, Boulagnon C, Bouland N *et al*: **Apelin expression deficiency in mice contributes to vascular stiffening by extracellular matrix remodeling of the aortic wall**. *Sci Rep* 2021, **11**(1):22278.
4. Axelsson AS, Mahdi T, Nenonen HA, Singh T, Hanzelmann S, Wendt A, Bagge A, Reinbothe TM, Millstein J, Yang X *et al*: **Sox5 regulates beta-cell phenotype and is reduced in type 2 diabetes**. *Nat Commun* 2017, **8**:15652.
5. El-Assaad W, Joly E, Barbeau A, Sladek R, Buteau J, Maestre I, Pepin E, Zhao S, Iglesias J, Roche E *et al*: **Glucolipotoxicity alters lipid partitioning and causes mitochondrial dysfunction, cholesterol, and ceramide deposition and reactive oxygen species production in INS832/13 ss-cells**. *Endocrinology* 2010, **151**(7):3061-3073.
6. Slove S, Lannoy M, Behmoaras J, Pezet M, Sloboda N, Lacolley P, Escoubet B, Bujan J, Jacob MP: **Potassium channel openers increase aortic elastic fiber formation and reverse the genetically determined elastin deficit in the BN rat**. *Hypertension* 2013, **62**(4):794-801.
7. Altunkaynak-Camca HO: **The Involvement of ATP-Sensitive Potassium Channels in the Nebivolol-Induced Relaxation of Endothelium-Intact Aorta Isolated from Rats**. *Middle Black Sea Journal of Health Science* 2020:201-206.
8. Vanelli G, Hussain SN, Aguggini G: **Glibenclamide, a blocker of ATP-sensitive potassium channels, reverses endotoxin-induced hypotension in pig**. *Exp Physiol* 1995, **80**(1):167-170.
9. Apkon M, Nerbonne JM: **Characterization of two distinct depolarization-activated K+ currents in isolated adult rat ventricular myocytes**. *J Gen Physiol* 1991, **97**(5):973-1011.
10. Mishra SK, Aaronson PI: **A role for a glibenclamide-sensitive, relatively ATP-insensitive K+ current in regulating membrane potential and current in rat aorta**. *Cardiovasc Res* 1999, **44**(2):429-435.
11. Shibukawa Y, Chilton EL, Maccannell KA, Clark RB, Giles WR: **K+ currents activated by depolarization in cardiac fibroblasts**. *Biophys J* 2005, **88**(6):3924-3935.
12. Rienecker KDA, Poston RG, Saha RN: **Merits and Limitations of Studying Neuronal Depolarization-Dependent Processes Using Elevated External Potassium**. *ASN Neuro* 2020, **12**:1759091420974807.
13. Wheeler DG, Barrett CF, Groth RD, Safa P, Tsien RW: **CaMKII locally encodes L-type channel activity to signal to nuclear CREB in excitation-transcription coupling**. *J Cell Biol* 2008, **183**(5):849-863.
14. Zou P, Liu L, Zheng L, Liu L, Stoneman RE, Cho A, Emery A, Gilbert ER, Cheng Z: **Targeting FoxO1 with AS1842856 suppresses adipogenesis**. *Cell Cycle* 2014, **13**(23):3759-3767.
15. Blaise S, Romier B, Kawecki C, Ghirardi M, Rabenoelina F, Baud S, Duca L, Maurice P, Heinz A, Schmelzer CE *et al*: **Elastin-derived peptides are new regulators of insulin resistance development in mice**. *Diabetes* 2013, **62**(11):3807-3816.
